# Impact of mutagenesis and lateral gene transfer processes in bacterial susceptibility to phage in food biocontrol and phage therapy

**DOI:** 10.3389/fcimb.2023.1266685

**Published:** 2023-09-28

**Authors:** Júlia López-Pérez, Jennifer Otero, Miquel Sánchez-Osuna, Ivan Erill, Pilar Cortés, Montserrat Llagostera

**Affiliations:** ^1^Departament de Genètica i de Microbiologia, Universitat Autònoma de Barcelona, Cerdanyola del Vallès, Spain; ^2^SK8 Biotech, Parc Científic de Barcelona, Barcelona, Spain; ^3^Department of Biological Sciences, University of Maryland, Baltimore, MD, United States; ^4^Departament Enginyeria de la Informació i de les Comunicacions, Universitat Autònoma de Barcelona, Cerdanyola del Vallès, Spain

**Keywords:** mutations, phage resistance, lateral gene transfer, phage interference, IncI1 plasmids, phage biocontrol, phage therapy

## Abstract

**Introduction:**

The emergence of resistance and interference mechanisms to phage infection can hinder the success of bacteriophage-based applications, but the significance of these mechanisms in phage therapy has not been determined. This work studies the emergence of *Salmonella* isolates with reduced susceptibility to a cocktail of three phages under three scenarios: i) *Salmonella* cultures (LAB), ii) biocontrol of cooked ham slices as a model of food safety (FOOD), and iii) oral phage therapy in broilers (PT).

**Methods:**

*S*. Typhimurium ATCC 14028 RifR variants with reduced phage susceptibility were isolated from the three scenarios and conventional and molecular microbiology techniques were applied to study them.

**Results and discussion:**

In LAB, 92% of *Salmonella* isolates lost susceptibility to all three phages 24 h after phage infection. This percentage was lower in FOOD, with 4.3% of isolates not susceptible to at least two of the three phages after seven days at 4°C following phage treatment. In PT, 9.7% and 3.3 % of isolates from untreated and treated broilers, respectively, displayed some mechanism of interference with the life cycle of some of the phages. In LAB and FOOD scenarios, resistant variants carrying mutations in *rfc* and *rfaJ* genes involved in lipopolysaccharide synthesis (phage receptor) were identified. However, in PT, the significant decrease of EOP, ECOI, and burst size observed in isolates was prompted by lateral gene transfer of large IncI1 plasmids, which may encode phage defense mechanisms. These data indicate that the acquisition of specific conjugative plasmids has a stronger impact than mutagenesis on the emergence of reduced phage-susceptibility bacteria in certain environments. In spite of this, neither mechanism seems to significantly impair the success of *Salmonella* biocontrol and oral phage therapy.

## 1 Introduction

The misuse and overuse of antibiotics in animal husbandry, human health care and agriculture have contributed to a substantial increase in antibiotic resistance rates worldwide. The spread of antimicrobial genes is achieved mainly by lateral gene transfer (LGT) events, not only among clinical pathogens but stemming also from commensal and environmental bacteria (von Wintersdorff et al., 2016; Sánchez-Osuna et al., 2019). The emergence of multi-drug resistant bacteria poses serious treatment limitations for a growing list of infectious diseases, increasing mortality rates as well as treatment costs, and urging the development of alternative therapies (European Antimicrobial Resistance Collaborators, 2022).

Bacteriophages have the potential to fulfill this need owing to several advantages when compared to antibiotics. For instance, phages self-replicate in a localized manner and can display a host range from broad to narrow, minimizing effects on commensal microbiota or tissues (de Jonge et al., 2019; Gordillo Altamirano and Barr, 2019). However, there are still some challenges that bacteriophages must overcome before being effectively and widely harnessed as antimicrobials (Górski et al., 2018). Primary among these hurdles is the unfolding of a broad range of anti-phage defense systems by bacteria, which could thwart the success of phage therapy approaches (Isaev et al., 2021a; Isaev et al., 2021b; Egido et al., 2022). These microbial strategies target different stages of the phage cycle: i) prevention of phage adsorption by masking/mutating surface receptors or secreting exopolysaccharides that hinder the access of phages to the surface of the host cell (Bertozzi Silva et al., 2016); ii) prevention of DNA injection by superinfection exclusion mechanisms (Bondy-Denomy et al., 2016; Rousset et al., 2022); iii) targeting the phage genome through mechanisms like restriction-modification systems (Tock and Dryden, 2005), bacteriophage exclusion (BREX) (Goldfarb et al., 2015), defense island associated mechanisms (DISARM) (Ofir et al., 2018) or prokaryotic Argonaute proteins (pAgos) (Makarova et al., 2009), as well as the adaptive immunity by CRISPR-Cas (clustered regularly interspaced palindromic repeats and CRISPR-associated proteins) system (Hille et al., 2018); iv) impairing cellular processes to block phage replication, transcription or translation via toxin-antitoxin, abortive infection systems (Dy et al., 2014; Lopatina et al., 2020) or broad-range anti-phage related systems based on retrons (Millman et al., 2020); and v) a plethora of putative defense systems identified recently and still not fully characterized (Doron et al., 2018; Millman et al., 2022).

Emergence of phage resistance against individual phages or phage cocktails has been reported *in vitro* (Pereira et al., 2016; Oechslin et al., 2017; Bai et al., 2019; Wright et al., 2019), where it is mediated mainly by mutations on genes encoding phage receptors. Similarly, phage resistance has been evidenced *in vivo* (Maura and Debarbieux, 2012; Schooley et al., 2017; Oechslin, 2018; De Sordi et al., 2019; Hernandez and Koskella, 2019; McCallin and Oechslin, 2019; Salazar et al., 2021) and although receptor mutations were also described as mechanism involved in some of them (Schooley et al., 2017), the mechanisms involved in the rest of studies have not been thoroughly investigated.

This study provides insights on the emergence of bacterial resistance and interference mechanisms against bacteriophages using *Salmonella enterica* serovar Typhimurium when exposed to a cocktail of three bacteriophages (UAB_Phi20, UAB_Phi78, and UAB_Phi87). Experiments were performed in three different scenarios: i) phage-infected *Salmonella* cultures (LAB); ii) biocontrol with the phage cocktail on cooked ham experimentally contaminated with *Salmonella*, as model of food matrices (FOOD), and iii) oral phage therapy applied to broilers infected with *Salmonella* in experimental farms (PT) (Colom et al., 2017). Our data reveal that the emergence of bacterial resistance to phages in LAB and FOOD was due to mutations in phage receptors, while acquisition of large plasmids encoding interference mechanisms of the multiplicative phage cycle drove bacterial resistance to phages in oral phage therapy. However, the emergence of bacterial resistance in food biocontrol and oral therapy settings did not significantly impair the success of phage treatments.

## 2 Materials and methods

### 2.1 Bacterial strains

*Salmonella enterica* serovar Typhimurium LB5000 (SGSC181; University of Calgary) was used to propagate and quantify the virulent bacteriophages used in this study and for mating studies. A *S*. Typhimurium ATCC 14028 spontaneous mutant resistant to rifampicin (Rif^R^), obtained by us, was used for the studies of the emergence of reduced phage-susceptibility of bacteria. Moreover, *S*. Typhimurium LT2 wild-type strain (University of Calgary), TA1537 strain (Dr. Bruce Ames, University of California, Berkeley), and a collection of lipopolysaccharide (LPS) mutants of *S*. Typhimurium LT2 (University of Calgary) were used to identify the possible receptors of phages UAB_Phi20, UAB_Phi78, and UAB_Phi87. Finally, the strain *Escherichia coli* DH5α was used for mutagenesis strategies (Clontech, Takara Bio, France) (**Supplementary Table S1**). The *S*. Typhimurium ATCC 14028 Rif^R^ strain and derived variants were cultured on Luria-Bertani (LB) or XLD Agar (Xylose-Lysine-Desoxycholate Agar; Becton Dickinson) media supplemented with Rifampicin (75 µg/mL). All the other bacterial strains were grown in LB broth or agar plates. In all cases, plates were incubated for 18 h at 37°C.

### 2.2 Bacteriophage propagation and titration

The bacteriophages studied belong to *Lederbergvirus* (UAB_Phi20, accession number: NC_031019), *Zendervirus* (UAB_Phi78, accession number: NC_020414) and *Felixounavirus* (UAB_Phi87, accession number: NC_027360) genera. These phages have been fully characterized and their genomes have been sequenced in previous studies (Bardina et al., 2012; Bardina et al., 2016).

Phage lysates were prepared following a previously described method (Colom et al., 2015) and filtered through 0.45 µm pore-size polyethersulfone (PES) membranes (Millex®-HP). The phage cocktail was prepared by mixing the UAB_Phi20, UAB_Phi78, and UAB_Phi87 bacteriophages with a ratio of 1:1:1, each phage at a titer of 3.3 log_10_ PFU/mL. The phage cocktail was further diluted with MgSO_4_ 10 mM to reach the desired concentration of experiments. Individual phages or phage cocktail titration was performed by plating ten-fold serial dilutions onto ATCC 14028 Rif^R^ LB plates using the double agar method (Kutter, 2009).

### 2.3 Isolation of *S*. Typhimurium ATCC 14028 Rif^R^ variants with reduced phage susceptibility

Detection of ATCC 14028 Rif^R^ isolates with reduced susceptibility to bacteriophages was studied in three different experimental conditions: i) *Salmonella* cultures infected with the phage cocktail (LAB), ii) cooked ham slices previously contaminated with *Salmonella* and treated with the phage cocktail (FOOD), and iii) oral phage therapy applied to broilers experimentally infected with *Salmonella* in experimental farms (PT) (Colom et al., 2017).

Infection of *Salmonella in vitro* cultures was performed as follows. A culture of ATCC 14028 Rif^R^ strain grown in LB medium at 37°C until an optical density at 550 nm (OD_550_) of 0.2 was infected with the phage cocktail (MOI of 1 PFU/CFU) and incubated for 24 h at 37°C. An uninfected culture was included as a control. Samples of both cultures were taken at 0, 4.5, 6.5, and 24 h, and appropriate ten-fold serial dilutions were plated on XLD-Rif (75 µg/mL). After 18 h of incubation at 37°C, around 50 colonies were randomly isolated on LB plates per time and type of culture for further studies.

For FOOD experiments, slices of packed cooked ham were purchased in a local supermarket, and the absence of *Salmonella* and bacteriophages capable of infecting the parental strain used in this study was verified through enrichment methods (Guttman et al., 2005). All the processes related to *Salmonella* contamination, phage treatment and drying of the ham slices were carried out in a level II biological safety cabinet (Telstar ™ Bio II Advance 4, Telstar Technologies S.L.). Firstly, slices of cooked ham were cut into 49 cm^2^ pieces using sterile material. To remove the remains of preservatives to ensure an efficient contamination with ATCC 14028 Rif^R^ strain, ham pieces were immersed in 0.9% NaCl (300 mL NaCl 0.9% for every 25 slices) and shaken at 50 r.p.m. for 5 min. Pieces of cooked ham were placed in trays covered with aluminum foil (previously disinfected with 70% ethanol and ultraviolet light for 15 min on each side) and dried in the cabin for 5 min (each side). One side of ham slices was contaminated with ATCC 14028 Rif^R^ strain by spraying a suspension in 0.9% NaCl [6.1 mL; 1 x 10^7^ colony-forming units (CFU)/mL] using an airbrush coupled to a compressor (Ventus Air-23) at a pressure of 1.5 bar. After 15 min of drying, pieces were treated by spraying with the phage cocktail in MgSO_4_ 10 mM (6.1 mL; 1 x 10^9^ PFU/mL). Control samples, untreated, were sprayed with the same volume of MgSO_4_ 10 mM. After an additional 15 min of drying, each slice of cooked ham was individually stored in Whirl-Pak filter bags (Nasco, Fort Atkinson, WI) at 4°C for 7 days. On days 0, 3, and 7, five pieces were taken, and 50 mL of buffered peptone water (BPW, Merck) was added to each bag. The contents were homogenized with a Bagmixer (Interscience) at maximum speed for 60 s. Determination of *Salmonella* concentration was done as previously described in XLD-Rif (75 µg/mL) (Colom et al., 2015). Finally, 240, 232, and 214 colonies from the plates of the control group on days 0, 3, and 7, respectively, were isolated on LB plates for further studies. Similarly, 235, 198, and 232 colonies were isolated at the same days from the treated group.

Concerning the PT experiment, *Salmonella* isolates came from a previous *in vivo* oral phage therapy study of *Salmonella* ATCC 14028 Rif^R^-infected commercial broilers and treated with the phage cocktail (Colom et al., 2017). Before infection, broilers were *Salmonella* and ATCC 14028 Rif^R^-specific phage free (Colom et al., 2017). Briefly, twenty-four cecum samples were collected on days 1, 8, and 15 after *Salmonella* infection from phage-treated and untreated group, respectively. After processing of samples (Colom et al., 2015), a total of 720 colonies were isolated from the control group (240 colonies per period) and 630 colonies from the bacteriophage-treated group (166 colonies on day 1, 225 on day 8, and 240 on day 15).

Randomly selected colonies were streaked on LB-Rif (75 µg/mL) and incubated for 18 h at 37°C. Afterwards, they were streaked on green plates (Chan et al., 1972) by three consecutive subcultures to ensure the absence of concomitant bacteriophages with the bacterial cells.

To determine the susceptibility to UAB_Phi78 phage, the isolates were grown in LB liquid medium in 96-well microtiter microplates (Sterilin™, Thermo Fisher Scientific, Massachusetts, USA) by incubation at 37°C with orbital shaking at 500 r.p.m (Kline; K3E model, Ovan, Barcelona, Spain) up to an absorbance at 550 nm (A_550_) of 0.12-0.15 (equivalent to 1 x 10^7^ CFU/mL). Then, cultures were infected with the phage at a MOI of 0.1. The plates were incubated at 37°C with shaking at 500 r.p.m, and growth was monitored by measuring the A_550_ every hour until 3 h, using the plate reader Sunrise (Tecan Trading AG, Switzerland). Moreover, uninfected clones were included as controls. Those isolates whose A_550_ increased over time in a similar way to the uninfected culture were considered to show reduced phage-susceptibility. In contrast, those whose A_550_ decreased over time were categorized as sensitive to phage. Strains ATCC 14028 Rif^R^ (sensitive to the three bacteriophages) and TA1537 (resistant to the three bacteriophages) were used as controls.

### 2.4 Spot assays

The sensitivity to UAB_Phi20 and UAB_Phi87 bacteriophages of all isolates showing reduced UAB_Phi78-susceptibility was determined by spot assay. Briefly, 10 µL of the bacteriophage lysate (1 x 10^6^ PFU/mL) were dropped onto the double layers of each isolate in LB agar plates and incubated at 37°C overnight (Kutter, 2009). Strains ATCC 14028 Rif^R^ (sensitive to the three bacteriophages) and TA1537 (resistant to the three bacteriophages) were used as controls.

### 2.5 Effect of phage infection on the bacterial growth kinetics

When necessary, the effect of phage infection on bacterial growth kinetics of the ATCC 14028 Rif^R^ variants was checked on liquid LB cultures as above described monitoring the A_550_ at 30-min or 1-h intervals after infection till 3.5 h, using the spectrophotometer Genesys 10S UV-Vis (Thermo Fisher Scientific, Massachusetts, USA). In addition, the concentration of viable cells (CFU/mL) was determined each time. Non-infected cultures of the studied strains were included as a control.

### 2.6 Efficiency of plating

Assessment of the efficiency of plating (EOP) was tested under the stationary phase of both target (ATCC 14028 Rif^R^ variants) and host (*S*. Typhimurium ATCC 14028 Rif^R^) strains. For this, the bacteria were cultured at 37°C for 18 h in LB medium, and 0.1 mL of the cultures were mixed with 0.1 mL of ten-fold serial dilutions of the appropriate phage lysate in LB molten agar (0.7%) and plated in LB agar by the double layer method. The plates were incubated at 37°C for 18 h and the number of PFU was counted. The EOP was calculated as described previously (Khan Mirzaei and Nilsson, 2015): average PFU on target bacteria/average PFU on host bacteria, and the standard deviation was also calculated for a minimum of three assays. For each phage, and according to the EOP value obtained, the variants were considered as high (EOP ≥0.5), medium (EOP < 0.5 but ≥0.1), low (EOP between 0.001 and 0.1) efficiency, and inefficient (<0.0001) (Khan Mirzaei and Nilsson, 2015).

### 2.7 Phage adsorption, burst size and efficiency of centre of infection

Phage adsorption to target bacteria and burst size of phages was determined as described previously (Kropinski, 2009; Kropinski, 2018). Likewise, center of infection (COI) and ECOI were determined as described previously (Moineau et al., 1993).

### 2.8 Characterization of *Salmonella* LPS profiles

The bacterial LPS was extracted following a protocol previously designed and kindly provided by Dr. J. Casadesús (Universidad de Sevilla) (Buendía-Clavería et al., 2003). The gels were digitally photographed with the Gel Doc XR system (Bio-Rad Laboratories, S.A).

### 2.9 Sequencing and analysis of *Salmonella* genomes

Total DNA was prepared using the Easy-DNA^TM^ kit (Invitrogen), following the manufacturer’s instructions. Sequencing was performed by Sistemas Genómicos S.L. (Sistemas Genómicos S.L, Valencia, Spain). The pool of the libraries was sequenced by paired-end sequencing (100 x 2) in an Illumina Hiseq 2500 sequencer. The sequencing resulted in around 9 million 101-nt long paired-end reads for each sample, with a mean Phred quality score > 30.

To find the putative mutations responsible for phage reduced susceptibility, the trimmed paired-end reads were aligned against the chromosome and pSLT plasmid sequences of *S*. Typhimurium ATCC 14028s (Genbank accession numbers: CP001363.1 and CP001362.1, respectively) using Geneious Prime 2020.0.4 (https://www.geneious.com) with up to 5 fine-tuning iterations. Single nucleotide polymorphisms (SNPs) calling was performed using the Geneious Prime software while larger insertions/deletions (≥ 5bp) were determined using Mauve Contig Mover (MCM) (Darling et al., 2010). Only insertions/deletions that were not found at the ends of the contigs (more than 20 bp away from the ends) and were absent in *S*. Typhimurium ATCC 14028 Rif^R^ parental strain were retained.

The existence of unused reads resulting from mapping assemblies revealed the presence of exogenous DNA to the *S*. Typhimurium ATCC 14028s chromosome or pSLT plasmid. To further investigate this acquired DNA, trimmed paired-end reads were re-assembled *de novo* using SPAdes v3.11.1 (with ‘careful -k 21,33,55,77’ settings) (Bankevich et al., 2012), and the scaffolds matching the genome or plasmid of *S*. Typhimurium 14028s through BLASTn (coverage>75%, e-value<1E-10) (Altschul et al., 1997) were removed from further analysis. To obtain the complete sequences of the acquired DNA, the retained scaffolds were combined with PLACNETw assemblies (Vielva et al., 2017) using Velvet (Zerbino, 2010) through Geneious Prime. Finally, complete plasmid assemblies were annotated using Prokka version 1.12 (Seemann, 2014) against the COG (Tatusov et al., 2000), HAMAP (Pedruzzi et al., 2015) and Pfam (Finn et al., 2016) databases and using their three best BLASTn complete plasmids hits as reference. The replicon sequences of each plasmid were determined using the PlasmidFinder 2.1 web-tool (Carattoli et al., 2014).

### 2.10 Construction of complemented strains

Construction of plasmids for complementation of mutations of target genes of *Salmonella* genomes was carried out by amplification of each wild-type gene of interest by PCR from the chromosome of ATCC 14028Rif^R^ using the appropriate primers (**Supplementary Table S2**). The primers carried homology for appropriate cloning at the 5’-end of the sequence and the PCR was done using the Phusion High-Fidelity DNA Polymerase (Thermo Scientific™). The resulting PCR products were purified with NzyGelpure kit (NZYTech, Lisboa, Portugal) and cloned into a previously linearized and dephosphorylated pUA1108 vector (**Supplementary Table S1**) via double digestion with NdeI and BamHI enzymes, as previously described (Mayola et al., 2014). The resulting ligation was used to transform competent *E. coli* DH5α cells by electroporation. Transformants were selected by growth on 50 µg/mL ampicillin plates. Plasmid DNA was extracted with a Plasmid DNA purification kit (NZYTech, Lisboa, Portugal) and the gene sequences were confirmed by sequencing (Macrogen).

The confirmed recombinant plasmids were introduced by electroporation in target *Salmonella* electrocompetent cells. Following selection of the transformants, the presence of cloned products was confirmed again by PCR and sequencing (Macrogen). As a control, the empty pUA1108 vector was transformed into the target bacteria under the same conditions.

### 2.11 Conjugative mating assays

Transference of phage interference mechanisms codified in acquired plasmids of PT variants was assessed by mating assays on a solid surface by adapting the method previously described (García-Quintanilla et al., 2008). *S*. Typhimurium LB5000 Str^R^ was used as recipient strain. The number of CFUs was recorded and the conjugation frequencies were calculated.

The susceptibility to the phages of the cocktail of four transconjugants of each matting experiment was assayed by spot assay, as described before (Kutter, 2009). Furthermore, the presence of acquired plasmids in such transconjugants was confirmed by conventional PCR using appropriate primers for sequences specific to each plasmid (**Supplementary Table S2**).

### 2.12 Introduction of kanamycin cassette on plasmids

Insertion of a kanamycin resistance cassette in the pUA1139 plasmid was performed using a λ-Red recombinase strategy (Datsenko and Wanner, 2000). Briefly, the kanamycin resistance cassette from pKD4 (**Supplementary Table S1**) was amplified by PCR using the primers with homology with the desired regions (**Supplementary Table S2**). The resulting linear PCR products were purified with NzyGelpure kit (NZYTech, Lisboa, Portugal), and were introduced by electroporation in the target bacteria carrying the plasmid pKOBEGA (Ap^R^; **Supplementary Table S1**). Transformant clones were selected in LB agar plates supplemented with kanamycin (50µg/mL). The kanamycin cassette insertion was confirmed by PCR and sequencing using the appropriate primers (**Supplementary Table S2**).

### 2.13 Plasmid curing methods

The CRISPR/Cas9-based genome editing method (Chen et al., 2018) was adapted to inactivate pUA1135 and pUA1136 plasmids identified in *S*. Typhimurium variants. The pCasPA plasmid encoding the Cas9 protein and the λ-Red recombination system and the pACRISPR plasmid with the BsaI site for cloning the spacers were used. First, the *bla* gene that codifies the beta-lactamase in the pACRISRPR plasmid was replaced by a spectinomycin (*spt*) gene obtained from pSET4s plasmid (Takamatsu et al., 2001). The resulting plasmid was named pUA1148 (**Supplementary Table S2**). The *spt* gene and pACRISPR (without the *bla* gene) were amplified by PCR using the Phusion polymerase (ThermoFisher Scientific) with appropriate primers (**Supplementary Table S2**). Then, the PCR products were assembled using the NEBuilder® HiFi DNA Assembly Master Mix (New England Biolabs, Inc) and transformed by electroporation in DH5α competent cells. The transformants were selected on LB agar plates supplemented with spectinomycin (100 μg/mL) and checked by conventional PCR.

The 20 nt sequence (spacer) targeting the *repA* gene of target plasmids was selected using the Find CRISPR Sites tool of the Geneious Prime software. For each CRISPR site identified, off-target binding sites in the *S*. Typhimurium genome or pSLT plasmid were also searched. The spacer with the highest activity and specificity scores was selected. The phosphorylated and annealed primers containing the spacer sequences were designed as described (Chen et al., 2018), and cloned into the pUA1148 using the NEB® Golden Gate Assembly Kit (BsaI-HF®v2; New England Biolabs, Inc) following the manufacturer’s instructions. The assembled product pUA1148+gRNA48 (**Supplementary Table S2**) was transformed by electroporation in DH5α competent cells and transformants were selected on LB agar plates supplemented with spectinomycin (100 μg/mL) and checked by PCR and sequencing (**Supplementary Table S2**). Afterwards, 1 µg of pCasPA plasmid was transformed by electroporation in *Salmonella* variants and the colonies were selected on LB agar plates supplemented with tetracycline (Tet; 17 μg/mL). Then, 100 ng of pUA1148+gRNA48 were transformed by electroporation into those *Salmonella* electrocompetent cells and the colonies containing pCasPA and pUA1148+gRNA48 were selected on LB agar plates with tetracycline (17 μg/mL) and spectinomycin (200 μg/mL). The elimination of the target plasmid was checked by PCR with suitable primers (**Supplementary Table S2**). Finally, the pCasPA and pUA1148+gRNA48 were cured by sucrose counter-selection (Chen et al., 2018).

To eliminate the pUA1139 plasmid, the curing method based on λ-Red mutagenesis and I-*Sce*I nuclease (de Moraes and Teplitski, 2015) was applied using the appropriate plasmids and primers (**Supplementary Tables S1, S2**). Briefly, one-step inactivation (Chaveroche et al., 2000; Datsenko and Wanner, 2000) was used to introduce the kanamycin resistance cassette (obtained from pKD4) and the restriction site for I-*Sce*I deleting gene *pUA1139_00007* from pUA1139 plasmid. At the same time, pUA1165 plasmid was constructed by removing the genes related to the λ-Red system from pKD46 and cloning the I-*Sce*I nuclease controlled by the tetracycline inducible promoter (P_tetA_) (synthesized at ATG:biosynthetics GmbH, Germany) into the NcoI restriction site using NEBuilder® HiFi DNA Assembly Master Mix (New England Biolabs, Inc) and transformed by electroporation in DH5α competent cells. The transformants were selected on LB agar plates supplemented with ampicillin (100 μg/mL) and checked by conventional PCR. Afterwards, 100 ng of pUA1165 were transformed by electroporation into the *Salmonella* IT2 variant containing the previously modified pUA1139 and made electrocompetent. Induction of I-*Sce*I nuclease was done using anhydrotetracycline (AHT) as described previously (de Moraes and Teplitski, 2015), and clones that were kanamycin sensitive were checked by PCR with suitable primers. Finally, pUA1165 was cured by temperature.

## 3 Results

### 3.1 Isolation of *S*. Typhimurium ATCC14028 Rif^R^ variants with reduced UAB_Phi78-susceptibility

**Figure 1:**
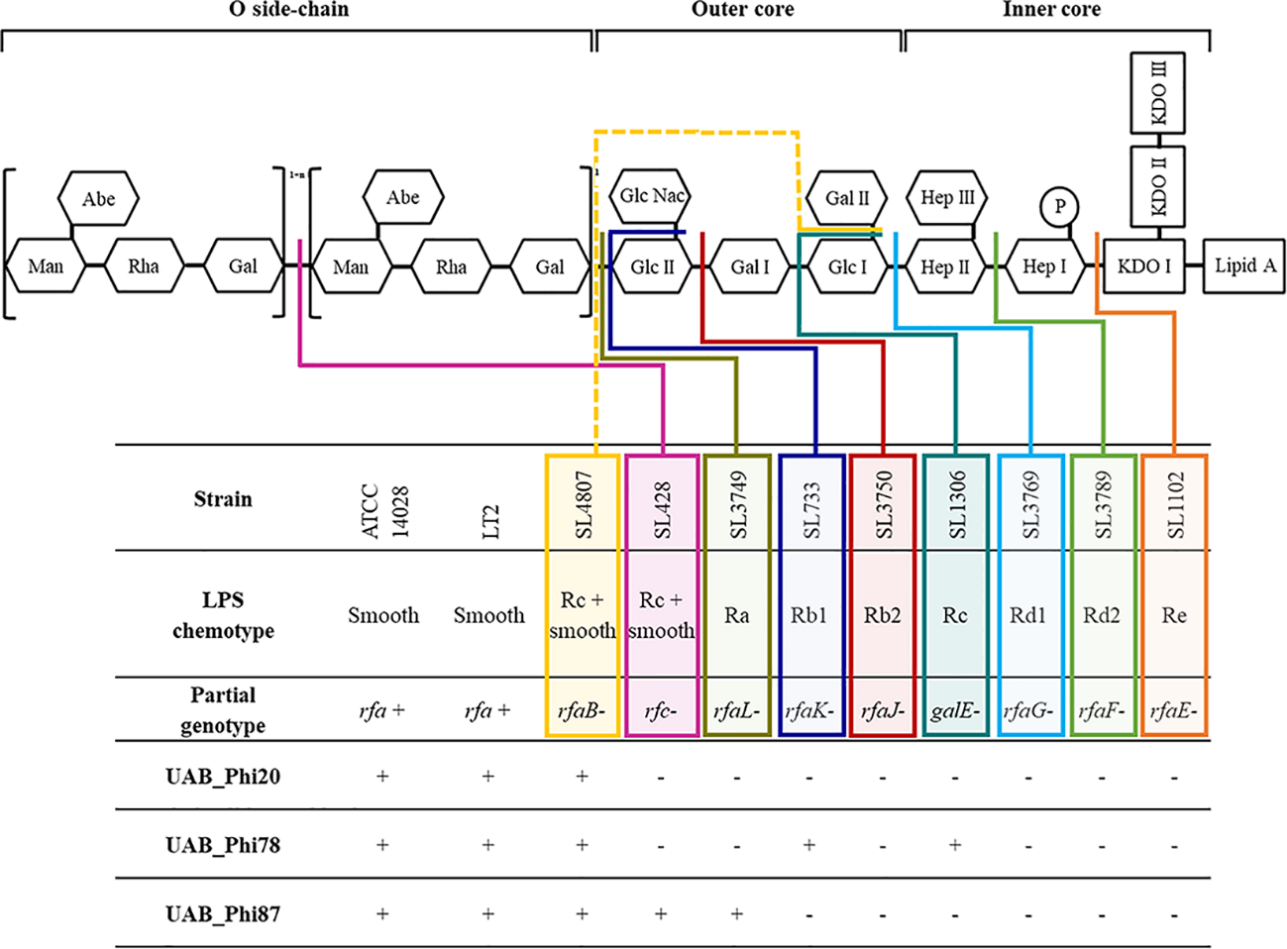
Scheme of *S*. Typhimurium LPS and detail of the infective capacity of bacteriophages UAB_Phi20, UAB_Phi78 and UAB_Phi87 against different LPS mutants of *S*. Typhimurium LT2 (based on Hitchcock et al., 1986). The different colored lines (solid and dashed) indicate the level of LPS impairment structure in the mutant strains +, lytic spot; -, no infection.

To study the emergence of bacterial resistance to phages in different settings, we isolated *S*. Typhimurium ATCC14028 Rif^R^ clones from the LAB, FOOD and PT experiments, and first identified variants that exhibited reduced susceptibility to UAB_Phi78 by monitoring absorbance at A_550_ of cultures infected with this phage. UAB_Phi78 was used for the initial screening due its fast multiplicate cycle (Bardina et al., 2012) and its ability to infect both smooth and rough *Salmonella* strains (**Figure 1**) (Bardina et al., 2016). From the LAB scenario, 200 and 197 isolates were randomly selected from uninfected and infected cultures, respectively, at different times after infection with the bacteriophage cocktail. All isolates from the uninfected cultures were sensitive to the UAB_Phi78 phage. However, 92% of isolates from the cocktail-infected culture displayed UAB_Phi78-reduced sensitivity 24 h after phage infection (**Table 1**).

**Table 1:** *S*. Typhimurium ATCC 14028 Rif^R^ variants with reduced UAB_Phi78-susceptibility isolated from uninfected and phage infected bacterial cultures (LAB).

Time (h)^a^	Uninfected		Infected	
	No. tested isolates	No. variants (%)	No. tested isolates	No. variants (%)
0	50	0 (0)	50	0 (0)
4	50	0 (0)	49	24 (49)
6.5	50	0 (0)	49	27 (55)
24	50	0 (0)	49	45 (92)
Total	200	0	197	96

**^a^** Time after infection of bacterial cultures (MOI=1) with the phage cocktail.

In the FOOD scenario, 686 and 665 isolates were obtained from 15 untreated and 15 treated cooked ham slices, respectively (**Table 2**). All isolates from the untreated cooked ham slices were sensitive to UAB_Phi78 phage, whereas 21 (3.2%) isolates from the treated slices showed reduced susceptibility to this phage.

**Table 2:** *S*. Typhimurium ATCC 14028 Rif^R^ variants with reduced UAB_Phi78-susceptibility isolated from untreated, and phage treated cooked ham slices (FOOD).

Time (days)^a^	Sample number	Untreated		Treated	
		No. tested isolates	No. variants (%)	No. tested isolates	No. variants (%)
0	1	48	0	48	0
	2	48	0	48	2
	3	48	0	43	0
	4	48	0	48	2
	5	48	0	48	1
Subtotal		240	0 (0)	235	5 (2.1)
3	1	47	0	48	1
	2	46	0	17	0
	3	46	0	43	2
	4	48	0	48	3
	5	45	0	42	0
Subtotal		232	0 (0)	198	6 (3.0)
7	1	48	0	46	0
	2	48	0	45	6
	3	30	0	48	2
	4	42	0	46	1
	5	46	0	47	1
Subtotal		214	0 (0)	232	10 (4.3)
Total		686	0 (0)	665	21 (3.2)

a Time after treatment of cooked ham slices with the phage cocktail (MOI=100).

In the PT scenario, 720 and 631 isolates were recovered from the ceca of untreated and treated broilers, respectively (Colom et al., 2017) (**Table 3**). Most of them were sensitive to the UAB_Phi78 phage, but 70 (9.7%) of the isolates from the untreated group and 21 (3.3%) from the group treated with the cocktail showed reduced susceptibility to this phage (**Table 3**).

**Table 3:** *S*. Typhimurium ATCC 14028 Rif^R^ variants with reduced UAB_Phi78-susceptibility isolated from untreated, and phage treated broiler chickens (PT).

Time (days)^a^	Animal number	Untreated group		Treated group	
		No. tested isolates	No. variants (%)	No. tested isolates	No. variants (%)
1	1	30	0	29	0
	2	30	0	30	0
	3	30	0	15	0
	4	30	0	30	0
	5	30	0	28	0
	6	30	0	26	0
	7	30	0	3	2
	8	30	0	5	0
Subtotal		240	0 (0)	166	2 (1.2)
8	1	30	0	15	0
	2	30	9	30	0
	3	30	1	30	1
	4	30	26	30	0
	5	30	9	30	0
	6	30	0	30	0
	7	30	0	30	0
	8	30	0	30	0
Subtotal		240	45 (18.7)	225	1 (0.4)
15	1	30	1	30	0
	2	30	6	30	0
	3	30	9	30	18
	4	30	0	30	0
	5	30	5	30	0
	6	30	0	30	0
	7	30	1	30	0
	8	30	3	30	0
Subtotal		240	25 (10.4)	240	18 (7.5)
Total		720	70 (9.7)	631	21 (3.3)

a An ATCC 14028 Rif^R^ strain suspension (10^7^ CFU/animal) was orally administered to animals at day 0. The three-phage cocktail (10^10^ PFU/animal) was orally administrated once daily from day -1 to day 7 of the infection (Colom et al., 2017). Time indicates the day of the ceca sampling for the bacteria isolation.

### 3.2 Molecular basis of reduced phage-susceptibility of *S*. Typhimurium ATCC 14028 Rif^R^ variants isolated from LAB and FOOD

**Figure 2:**
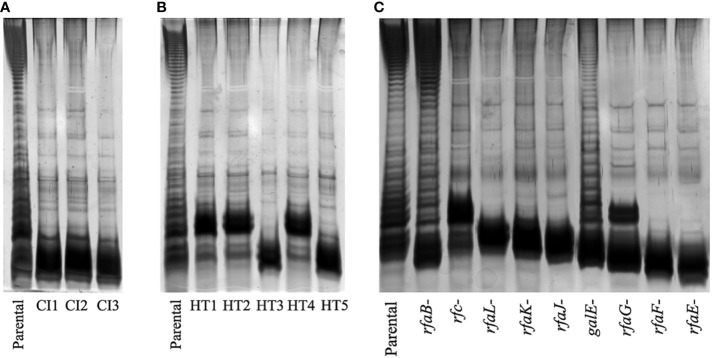
LPS electrophoretic profiles of the variants isolated from **(A)** LAB, **(B)** FOOD, and **(C)** LPS mutants of *S*. Typhimurium LT2 strain. The LPS electrophoretic profile of the ATCC 14028 Rif^R^ parental strain is also shown as control.

All variants with reduced UAB_Phi78-susceptibility isolated from LAB and FOOD scenarios were insensitive to UAB_Phi78 infection and their efficiency of plating (EOP) was zero. Data from the spot test analysis to the two other phages of the cocktail showed that the 96 variants isolated from treated LAB samples (**Table 1**) were also insensitive to infection by the other phages of the cocktail. In the case of the 21 variants isolated from treated FOOD samples (**Table 2**), 12 were also insensitive to UAB_Phi20 and 9 were insensitive to the three phages of the cocktail. Our initial hypothesis was that these variants were resistant to phage infection due to changes in their phage receptors, which are located in the LPS of the outer membrane. To study this, eight of these variants were selected (**Table 4**), and their LPS electrophoretic profile was assessed and compared with those of the parental strain *S*. Typhimurium ATCC 14028Rif^R^ and the *S*. Typhimurium LT2 LPS mutants (**Supplementary Table S1**). Results showed that the LPS electrophoretic profile of five variants isolated from both LAB (CI1, CI2 and CI3) and FOOD (HT3 and HT5) was similar to those of the *rfaL*-, *rfaK*-, *rfaJ*-, and *rfaI*- rough mutants (**Figure 2**). On the other hand, the HT1, HT2 and HT4 variants from FOOD showed a LPS electrophoretic profile resembling that of the *rfc*- rough mutant (**Figure 2**).

**Table 4:** Selected reduced UAB_Phi78-susceptibility *S*. Typhimurium ATCC 14028 Rif^R^ variants isolated from LAB and FOOD for further characterization.

Scenario^a^	Group	Time^b^	Variant name^c^	Phenotype^d^	
				UAB_Phi20	UAB_Phi87
LAB	Infected	4 h	CI1	–	–
		6.5 h	**CI2**	–	–
		24 h	**CI3**	–	–
FOOD	Treated	0	**HT1**	–	+
		3 d	HT2	–	+
			HT3	–	–
		7 d	HT4	–	+
			**HT5**	–	–

a LAB, laboratory cultures; FOOD, cooked ham slices.

b Time indicates the day (d) or hour (h) of variants isolation after phage cocktail application in FOOD and LAB, respectively.

c Variants selected for further genome sequencing and analysis are highlighted in bold.

d Determined by spot test. +, clear plaques; -, no plaques.

To further explore the hypothesis that the observed resistance was due to changes in phage receptors, we sequenced the genomes of the variants indicated in **Table 4** and that of the parental strain ATCC 14028 Rif^R^, and we mapped their sequencing reads to the reference ATCC 14028s genome (GenBank CP001363.1). Results revealed several point mutations in the genomes of all variants and the parental strain when compared to the NCBI reference genome (**Supplementary Table S3**). To test our hypothesis, we focused on identifying mutations in genes involved in LPS biosynthesis present in the variants but absent in the parental strain genome. In this regard, we found that the genomes of the CI2 and CI3 variants from LAB and the HT5 variant from FOOD, which display an identical phage sensitivity phenotype, contained point mutations in the *rfaJ* gene. Specifically, the CI2 and CI3 variants displayed a G→T transversion at position 910 of this gene, while the genome of the HT5 variant had an adenine deletion at position 742 of the *rfaJ* gene (**Table 5**). Furthermore, after PCR amplification and sequencing of the *rfaJ* gene, we found that the CI1 variant had the same G→T transversion identified in CI2 and CI3, while an adenine deletion at position 715 was detected in the HT3 variant (**Table 5**). All these point mutations give rise to a premature ochre stop codon (UAA) in the *rfaJ* gene.

**Table 5:** Mutations identified in the *rfc* and *rfaJ* genes of selected *S*. Typhimurium ATCC 14028 Rif^R^ variants isolated from LAB and FOOD.

Scenario^a^	Variant name	Gene	Type of mutation^b^	Phenotype^c^		
				UAB_Phi78	UAB_Phi20	UAB_Phi87
LAB	CI1	*rfaJ*	G/C to T/A at position 910	–	–	–
	**CI2**		G/C to T/A at position 910	–	–	–
	**CI3**		G/C to T/A at position 910	–	–	–
FOOD	HT3	*rfaJ*	(- A) at position 715	–	–	–
	**HT5**		(- A) at position 742	–	–	–
	**HT1**	*rfc*	Deletion of 2,557 bp including the *rfc* gene	–	–	+
	HT2		C/G to A/T at position 219	–	–	+
	HT4		Deletion of 2,557 bp including the *rfc* gene	–	–	+

a LAB, laboratory cultures; FOOD, cooked ham slices.

b Mutations detected in variants highlighted in bold were determined by genome sequencing. Mutations in the other variants were identified by PCR amplification of *rfaJ* or *rfc* genes and DNA sequencing.

c Determined by spot test. +, clear plaques; -, no plaques.

**Figure 3:**
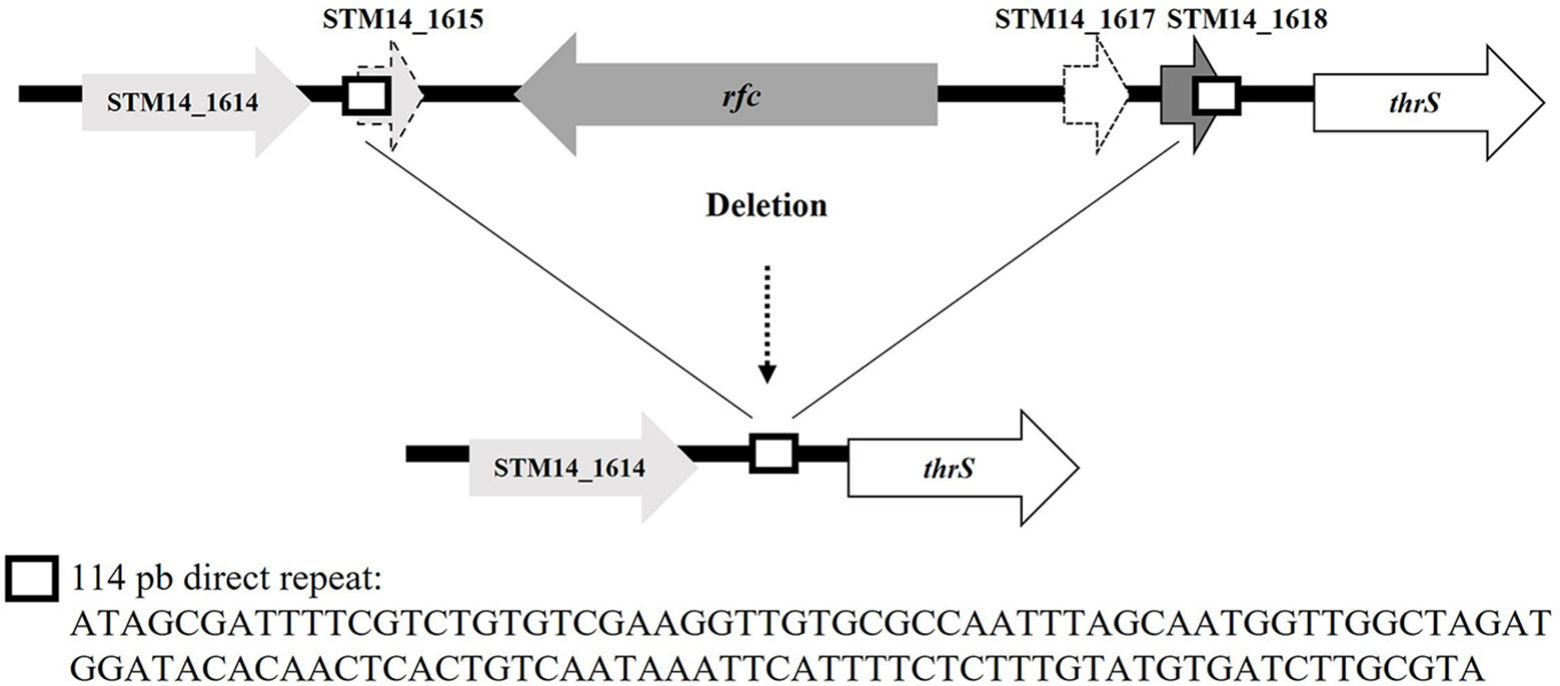
Deletion found in the *rfc* gene in variants HT1 and HT4 isolated from FOOD. The sequence of direct repeats is shown.

On the other hand, the genome sequence of the HT1 variant from FOOD showed a deletion of 2,557 bp that encompassed the *rfc* gene. In the parental strain genome, this region is flanked by two direct repeats (DR) of 114 bp located upstream of the STM14_1615 gene (CP001363.1: 1,419,477–1,419,592) and downstream of the STM14_1618 gene (CP001363.1: 1,422,035–1,422,148) (**Figure 3**). The HT1 variant deletion involved the STM14_1615, *rfc*, STM14_1617, and STM14_1618 genes and the loss of one DR (**Figure 3**). Furthermore, by using PCR amplification and DNA sequencing, we searched for mutations in this DNA region in the genomes of HT2 and HT4 variants. We identified the same deletion in the HT4 variant and a C→A transversion at position 219 of the *rfc* gene of the HT2 variant giving rise to a premature ochre stop codon (UAA) (**Table 5**).

Finally, we demonstrated that complementation *in trans* of the *rfc* and *rfaJ* wild-type genes in the variants that presented mutations in these genes restored both the sensitivity to phages and LPS profiles seen in the parental strain.

### 3.3 Molecular basis of reduced phage-susceptibility of *S*. Typhimurium ATCC 14028 Rif^R^ variants isolated from PT

In the PT scenario, the A_550_ of cultures for *S*. Typhimurium ATCC 14028 Rif^R^ variants with reduced UAB_Phi78-susceptibility (**Table 3**) remained almost constant or increased after infection with the UAB_Phi78 phage, although their absorbances were lower than that of the non-infected parental strain (**Supplementary Figure S1**). The number of viable cells declined, especially in one of the two variants, but to a much lesser extent than observed in the UAB_Phi78-infected parental strain. These data suggested that phage infection could cause cell death of these variants, but with limited lysis activity. Spot test analyses of the two other cocktail phages revealed that, of the 70 variants with reduced susceptibility to UAB_Phi78 isolated from the untreated group (**Table 3**), 62 were insensitive to the other two phages of the cocktail (UAB_Phi20 and UAB_Phi87), and 8 only to the UAB_Phi87 phage. In contrast, all 21 variants isolated from the treated group (**Table 3**) were insensitive to UAB_Phi87 phage, but sensitive to UAB_Phi20 phage. Sixteen variants from untreated and treated animal groups were selected according to their susceptibility profile to the cocktail phages and to the date of isolation (**Table 6**), and their LPS electrophoretic profiles were examined. We observed no differences between the electrophoretic profiles of these variants and that of the parental strain (**Supplementary Figure S2**), suggesting that they have functional phage receptors. Consequently, we hypothesized that these variants might have some mechanism of interference with the multiplicative cycle of the phages. Therefore, seven of these variants (highlighted in bold in **Table 6**) were chosen for an in-depth study.

**Table 6:** Selected reduced UAB_Phi78-susceptibility *S*. Typhimurium ATCC 14028 Rif^R^ variants isolated from PT.

Scenario^a^	Group	Animal^b^	Time^c^	Variant name^d^	Phenotype^e^	
			(days)		UAB_Phi20	UAB_Phi87
PT	Untreated	2	8	IC1	–	–
		3		IC2	+	–
		4		**IC3**	–	–
		5		IC4	–	–
		5		**IC5**	–	–
		1	15	**IC6**	+	–
		2		IC7	+	–
		2		**IC8**	+	–
		3		IC9	–	–
		5		**IC10**	–	–
		7		IC11	–	–
		8		IC12	–	–
	Treated	7	1	IT1	+	–
		3	8	**IT2**	+	–
		3	15	**IT3**	+	–
		3		IT4	+	–

a PT, phage therapy.

b Indicates the animal number from which each variant was isolated.

c Time indicates the day post-infection the ceca were obtained for the bacteria isolation.

d Variants selected for further studies to determine the adsorption kinetic, diverse infection parameters, and genome sequencing of the phages are highlighted in bold. All variants were isolated from different broilers except the pairs of variants IC4-IC5 and IT3-IT4 obtained each from the same animal.

e Determined by spot test. +, clear plaques; -, no plaques.

Firstly, we proceeded to determine the adsorption constant (*K*) and various infection parameters [efficiency of plating (EOP), efficiency of center of infection (ECOI) and burst size] of the three phages. The data obtained revealed that all seven variants had functional receptors for both UAB_Phi78 and UAB_Phi20 phages since *K* values of both phages in the variants were like those for the parental strain (**Table 7**). Regarding the EOP, ECOI and burst size parameters of both phages, variants were divided into two groups. One group, encompassing the IC3, IC5 and IC10 variants, was insensitive to these phages with EOP and ECOI values of 0 (**Table 7**). The other group, which included IC6, IC8, IT2 and IT3 variants, displayed a medium production efficiency [EOP values between 0.1 and 0.5 (Khan Mirzaei and Nilsson, 2015)] for both phages, with the exception of the IT3 variant, considered as low production efficiency [EOP values between 0.001 and 0.1 (Khan Mirzaei and Nilsson, 2015)] for UAB_Phi78 phage, and the IC6 variant, which achieved high production [EOP values ≥ 0.5 (Khan Mirzaei and Nilsson, 2015)] for the UAB_Phi20 phage. For both phages, ECOI values of the variants in this group were < 0.5 and burst sizes were lower than those obtained in the parental strain (**Table 7**). On the other hand, although we could determine that the UAB_Phi87 phage adsorbs to the parental strain with a low *K* value (2.3E-9 ± 7.5E-10 mL/min), the slopes of adsorption curves in these variants were lower than that of the parental strain, and the *K* values could not be calculated as described previously (Kropinski, 2009). In addition, no plaques were observed when this phage was titered on these variants.

**Table 7:** Parameters of phages UAB_Phi78 and UAB_Phi20 on reduced phage-susceptibility variants isolated from PT.

Parental strain Variant name	UAB_Phi78				UAB_Phi20			
	*K* ^a^ (ml/min)	EOP^b^	ECOI^c^	Burst size (PFU/cell)	*K* ^a^ (ml/min)	EOP^b^	ECOI^c^	Burst size (PFU/cell)
ATCC 14028 Rif^R^	3.9E-8 ± 2.7E-8	1.0	1.0	98.16 ± 2.6	1.1E-8 ± 5.2E-9	1.0	1.0	189.2 ± 9.6
IC6	3.1E-8 ± 1.9E-8	0.2 ± 0.1	0.3 ± 0.1	9.48 ± 1.1	6.7E-9 ± 3.4E-9	0.6 ± 0.1	0.3 ± 0.0	142.9 ± 7.4
IC8	2.4E-8 ± 1.0E-8	0.2 ± 0.0	0.3 ± 0.0	10.2 ± 1.1	7.4E-9 ± 8.2E-9	0.4 ± 0.1	0.2 ± 0.1	26.0 ± 2.2
IT2	3.3E-8 ± 2.4E-8	0.1 ± 0.0	0.3 ± 0.1	6.4 ± 1.8	1.3E-8 ± 8.2E-9	0.4 ± 0.1	0.2 ± 0.1	83.5 ± 6.9
IT3	2.1E-8 ± 1.7E-8	0.01 ± 0.0	0.2 ± 0.1	22.4 ± 0.3	7.1E-9 ± 3.5E-9	0.4 ± 0.1	0.2 ± 0.0	34.5 ± 2.6
IC3	7.5E-9 ± 4.6E-9	0.0	0.0	ND	3.7E-9 ± 2.8E-9	0.0	0.0	ND
IC5	5.4E-7 ± 4.1E-8	0.0	0.0	ND	1.2E-8 ± 7.1E-9	0.0	0.0	ND
IC10	5.7E-9 ± 3.2E-9	0.0	0.0	ND	5.0E-9 ± 2.3E-9	0.0	0.0	ND

For all the parameters, the mean of at least two independent experiments ± standard deviation is shown.

ND, not determined due to the inefficient infection of phage.

a Adsorption rate constant (*K*).

b Efficiency of plating (EOP).

c Efficiency of centre of infection (ECOI).

Altogether, the results above confirmed our hypothesis that the variants isolated from PT with reduced sensitivity to phages must have some mechanism of interference that affects processes involved in phage multiplication in bacterial cells. To elucidate this, the genomes of these seven variants were sequenced. Analysis of these genome sequences revealed no point mutations or indels involving known genes related to phage defense mechanisms when compared to the genome sequence of the parental strain (**Supplementary Table S3**). However, analysis of unmapped Illumina reads demonstrated that all these variants carried several plasmids in addition to the pSLT plasmid, known to be present in the serovar Typhimurium of *S*. *enterica* (Jones et al., 1982). Sequencing determined that all variants carried a large conjugative IncI1α plasmid (~96-110 Kb) (**Table 8**). IC3, IC5, and IC10 variants harbored the pUA1135 plasmid, encoding a CTX-M-14 beta-lactamase. IC6 and IC8 variants carried the pUA1136 plasmid, encoding a TEM-1 beta-lactamase, and IT2 and IT3 variants contained the pUA1139 plasmid, which did not encode any known antibiotic-resistance mechanism (**Table 8**). Furthermore, some of these variants carried additional lower-size plasmids from different incompatibility groups (**Table 8**).

**Table 8:** Characteristics of plasmids identified in reduced phage-susceptibility variants isolated from PT.

Variant name	Plasmid	Size (pb)	Replicon (% identity)^a^	Antibiotic resistance	Genbank accession number
IC3	pUA1135	96,685	IncI1 (100)	CTX-M-14	MW590592
	pUA1144	4,612	NF	–	MW655531
	pUA1145	3,257	Col440I (93.8)	–	MW655532
IC5	pUA1135	96,685	IncI1 (100)	CTX-M-14	MW590592
IC10	pUA1135	96,685	IncI1 (100)	CTX-M-14	MW590592
	pUA1144	4,612	NF	–	MW655531
IC6	pUA1136	104,489	IncI1 (99.3)	TEM-1	MW655523
IC8	pUA1136	104,489	IncI1 (99.3)	TEM-1	MW655523
	pUA1138	31,244	IncX4 (100)	–	MW655525
IT2	pUA1139	110,041	IncI1 (100)	–	MW655526
IT3	pUA1139	110,041	IncI1 (100)	–	MW655526
	pUA1141	38,709	IncX4 (86.1)	–	MW655528
	pUA1142	5,463	Col156 (94.8)	–	MW655529

a Identity obtained with PlasmidFinder 2.1 (80% of nucleotide identity threshold); NF, not found.

**Figure 4:**
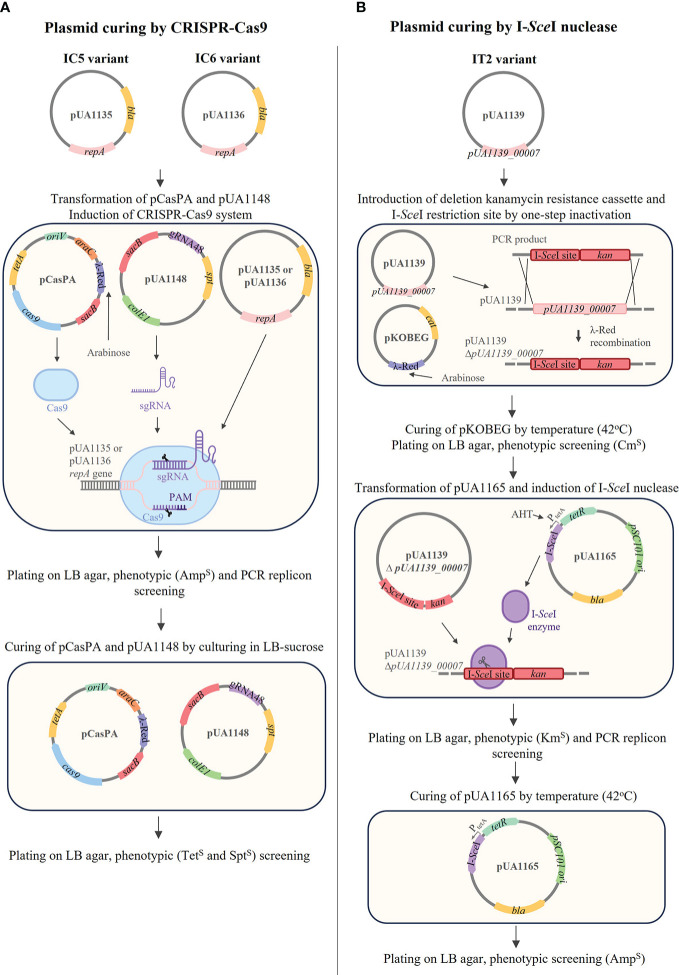
Procedures for curing plasmids pUA1135, pUA1136 and pUA1139 from IC5, IC6, and IT2 variants, respectively. **(A)** pUA1135 and pUA1136 were cured using CRISPR-Cas9 targeting *repA* gene. First, pCasPA and pUA1148 were transformed into each variant, and the CRISPR-Cas9 system was induced by arabinose. Cured clones were checked by phenotypic screening (Amp^S^) and PCR amplifying the *repA* gene. Plasmids pCasPA and pUA1148 were cured in the presence of sucrose, and cured clones were checked by phenotypic screening (Tet^S^ and Spt^S^). **(B)** pUA1139 was cured by λ-Red mutagenesis and I-*Sce*I nuclease. First, the PCR product containing I-*Sce*I restriction site and the Km^R^ cassette was transformed into the variant containing pKOBEG, deleting the gene *pUA1139_00007*, by one-step inactivation. pKOBEG was cured by culturing at 42°C and clones were screened for Cm^S^. Then, pUA1165 was transformed into the modified cell and I-*Sce*I nuclease was induced by the addition of AHT. Cured clones were checked by phenotypic screening (Km^S^) and PCR with primers specific for the *repA* gene. pUA1165 was cured by culturing at 42°C and clones were screened for Amp^S^.

Since the IC5, IC6, and IT2 variants only carried, respectively, the pUA1135, pUA1136, and pUA1139 large plasmids, we hypothesized that these plasmids encode interference mechanisms. To demonstrate this, these variants were used as donors in conjugation experiments, using the phage-sensitive and streptomycin-resistant *S*. Typhimurium LB5000 strain as a recipient. Transconjugant strains receiving the pUA1135 and pUA1136 plasmids were selected on streptomycin and ampicillin plates, and streptomycin and kanamycin plates were used for transconjugants for the pUA1139 plasmid, to which a kanamycin resistance cassette had been previously introduced by one-step inactivation (Datsenko and Warner, 2000). The mating frequencies were 5.0E-3, 4.7E-3, and 9.0E-4 for the pUA1135, pUA1136, and pUA1139 plasmids, respectively. The presence of large plasmids in four transconjugants of each mating was confirmed by PCR, using specific primers for each plasmid (**Supplementary Table S1**). EOP and ECOI values for both UAB_Phi20 and UAB_Phi78 phages were determined for one transconjugant of each mating (**Table 9**). Results indicated that the acquisition by conjugation of the pUA1135 plasmid gave rise to transconjugants insensitive to both UAB_Phi20 and UAB_Phi78 phages. In addition, transconjugants that acquired the pUA1136 and pUA1139 plasmids also exhibited a reduced sensitivity to UAB_Phi20 and UAB_Phi78 phages, with EOP and ECOI values quite similar to those of the bacterial donors (**Table 9**). In this regard, it must be considered that the genetic backgrounds of the donor and recipient strains were different since the donor strain derived from *S*. Typhimurium ATCC 14028 Rif^R^, and the recipient was *S*. Typhimurium LB5000. Concerning phage UAB_Phi87, the transconjugants exhibited a reduced sensitivity to this phage, with EOP values around 0.1-0.2 and lower size plaques compared to the ones observed on the parental strain (LB5000) (**Table 9**). To corroborate this, plasmids pUA1135, pUA1136, and pUA1139 were subsequently cured from IC5, IC6 and IT2 variants, respectively (**Figure 4**). Their loss entailed the recovery of sensitivity to both UAB_Phi20 and UAB_Phi78 phages, as evidenced by EOP values like those of *S*. Typhimurium ATCC 14028 Rif^R^ However, sensitivity to the UAB_Phi87 phage was not fully recovered, since lower EOP values (≤ 0.1) than those of the parental strains were observed.

**Table 9:** Values of EOP and ECOI of UAB_Phi78, UAB_Phi20 and UAB_Phi87 phages on both donor and transconjugants.

Donor							Transconjugant						
Variant name	Plasmid	UAB_Phi78		UAB_Phi20		UAB_Phi87	Plasmid^b^	UAB_Phi78		UAB_Phi20		UAB_Phi87	
		EOP^a^	ECOI^a^	EOP^a^	ECOI^a^	EOP^a^		EOP^c^	ECOI^c^	EOP^c^	ECOI^c^	EOP^c^	
IC5	pUA1135	0.0	0.0	0.0	0.0	0.0	pUA1135	0.0	0.0	0.0	0.0	0.2± 0.0	
IC6	pUA1136	0.2 ± 0.1	0.3 ± 0.1	0.6 ± 0.1	0.3 ± 0.03	0.0	pUA1136	0.2 ± 0.1	0.3 ± 0.0	0.7 ± 0.0	0.8 ± 0.0	0.1± 0.0	
IT2	pUA1139	0.1 ± 0.0	0.3 ± 0.1	0.4 ± 0.1	0.2 ± 0.1	0.0	pUA1139	0.1 ± 0.0	0.6 ± 0.1	0.2 ± 0.0	0.5 ± 0.1	0.1± 0.0	

a The values were calculated compared to the parental strain *S*. Typhimurium ATCC14028 Rif^R^.

b The presence of plasmid was confirmed by PCR amplification.

c The values were calculated compared to the *S*. Typhimurium LB5000 because it was used as the recipient strain.

## 4 Discussion

The increased interest in the application of bacteriophages as therapeutic tools raises the question of whether the emergence of bacteria with reduced phage-susceptibility could jeopardize the effectiveness of this antibacterial strategy. In certain applications, environmental conditions such as lower temperatures, variable resource availability, low phage-to-bacteria ratio and competing surrounding microbiota could influence the emergence of phage-resistant bacteria. Other aspects, such as the ability of bacteriophages to co-evolve with the host, overcoming both phage resistance and bacterial defense mechanisms, the bacterial fitness cost and compromised virulence that entails the development of resistance, and the use of bacteriophage cocktails preventing the development of resistance, are also issues that need to be addressed (Levin and Bull, 2004; Oechslin, 2018).

The present work studies the emergence of bacterial variants with reduced susceptibility to bacteriophages, using *Salmonella* and a cocktail composed by three bacteriophages (UAB_Phi20, UAB_Phi78, and UAB_Phi87) in three different environmental conditions: *in vitro* cultures (LAB), cooked ham slices (FOOD), and oral phage therapy in broilers (PT).

Our experimental approach was to first isolate *Salmonella* variants with reduced susceptibility to the UAB_Phi78 phage due to its dual adsorption ability and subsequently determine their susceptibility to the other two phages. UAB_Phi78 phage is capable of infecting both smooth bacteria and specific rough mutants (**Figure 1**) because it presumably has two functional receptors as previously described for the SP6 phage (Tu et al., 2017). The tail spike proteins (TSP) would interact with the O-antigen in the infection of smooth bacteria, and the phage tail itself would interact with some component of the inner LPS core when infecting some rough mutants (Tu et al., 2017). This assumption for UAB_Phi78 phage is based on results in LPS mutant strains (**Figure 1**) and the high sequence identity (≥ 96%) of both its TSP and tail fiber proteins to those of SP6 phage (Bardina et al., 2016). Furthermore, as UAB_Phi78 showed a faster multiplicative cycle than the other two phages of the cocktail, this could favor an earlier selection of variants with reduced sensitivity to this phage.

Our results highlight a much higher percentage of isolation of variants with reduced UAB_Phi78-susceptibility in the LAB setting than in either the FOOD or PT scenarios. Furthermore, mutagenesis seems to be the biological process involved in the emergence of LAB and FOOD resistance while, in suitable conditions, phage interference mechanisms encoded in large conjugative plasmids surface in oral PT.

Results from the LAB setting demonstrated that the percentage of isolation of variants with reduced UAB_Phi78 susceptibility increased over time, achieving 92% after 24 h of the infection of cells with the cocktail (**Table 1**). The EOP of UAB_Phi78 and the other two phages of the cocktail was zero in all these isolates. Bacteriophage-insensitive mutants have also been encountered by other authors in *in vitro* conditions (Pereira et al., 2016; Oechslin et al., 2017; Bai et al., 2019; Wright et al., 2019). Genome sequencing and subsequent trans-complementation assays of three (CI1, CI2 and CI3) of the LAB variants (**Table 5**) pointed to mutations in the receptors of the three phages. These mutations generated a premature stop codon at position 910 in the *rfaJ* gene, which encodes a UDP-glucose:(Galactosyl) LPS α-1,2-glucosyltransferase involved in the addition of α-l, 2-linked glucose II residues to the LPS core (Schnaitman and Klena, 1993) (**Figure 1**), resulting in insensitivity to the three phages. Consistent with this finding, the LPS electrophoretic profiles of these variants resembled that of the *S*. Typhimurium *rfaJ*- rough mutant (**Figure 2**) with identical phage insensitivity patterns (**Figure 1**). Moreover, it should be noted that CI1, CI2 and CI3 variants were isolated, respectively, at 4, 6.5 and 24 h following bacterial cultures infection. This would suggest a clonal relationship among these variants, with this higher-fitness clone being the most abundant in the cultures after 24 h of incubation.

On the other hand, the percentage of variants showing reduced UAB_Phi78-susceptibility in cooked ham slices treated with the cocktail and maintained at 4°C (FOOD) increased only slightly over time, with values of 2.1%, 3.0%, and 4.3% at 0, 3 and 7 days, respectively (**Table 2**). The low percentages obtained agree with those observed in different biocontrol studies applying individual or phage cocktails on meat where reduced phage-susceptibility was rarely detected (Abuladze et al., 2008; Hong et al., 2016; Tomat et al., 2018). The low percentages of variants with reduced phage-susceptibility obtained in this scenario compared to LAB could be attributed to a low initial bacterial concentration in FOOD, the solid surface of meat inhibiting bacterial motility, limiting phage selective pressure and the spread of resistant variants, as well as the incubation temperature (4°C), which significantly decreases the *Salmonella* metabolic activity and bacteriophage multiplication. It should be noted that zero-day time (**Table 2**) corresponds to the initial day of storing cooked ham slices at 4°C after phage treatment. Therefore, the identification of variants with reduced UAB_Phi78-susceptibility on treated samples at this time suggested that they were possibly present in the *Salmonella* culture used to contaminate the cooked ham slices and were selected by phage predation pressure during treatment and storage (15 min), because no such variants were identified in control samples after the same period (**Table 2**). Not all variants with reduced UAB_Phi78-susceptibility displayed reduced susceptibility to the other two phages. Twelve of them were only insensitive to the UAB_Phi20 phage, while nine were insensitive to the other two phages. Genome analysis and trans-complementation determined that point mutations producing a truncated RfaJ protein were responsible for the insensitivity to the three phages in some variants (HT3 and HT5) (**Table 5**). In contrast, the loss of the O-antigen ligase, encoded by the *rfc* gene and required for polymerization of multiunit O-antigen molecules (Schnaitman and Klena, 1993) (**Figure 1**), prevented the infection of the variants HT1, HT2 and HT4 by both UAB_Phi78 and UAB_Phi20 phages, but not by UAB_Phi87 phage (**Table 5**). This can be due to the *rfc* mutants displaying the N-acetylglucosamine residue of the terminal glucose II of the LPS core, necessary for UAB_Phi87 adsorption, but losing the multiunit O-antigen that allows UAB_Phi20 and UAB_Phi78 phage adsorption to the Rc+ smooth strains, as it occurred in *S*. Typhimurium SLA28 strain (**Figure 1**). These results were in agreement with the infection profiles of the three bacteriophages for different LPS mutants of *S*. Typhimurium LT2 (**Figure 1**) and with the LPS electrophoretic profile of these variants (**Figure 2**). In contrast to LAB, no clonal relationship can be inferred among the analyzed variants of FOOD, except for HT1 and HT4 (**Table 5**), which present the same mutation in the *rfc* gene. Since the mutational events that gave rise to RfaJ and Rfc truncated proteins were found in different cooked ham slices, we believe these could be the most frequent mutational events or those that conferred the highest fitness to *Salmonella* cells selected by the phage treatment. It should be noted that the involvement of LPS or other external structures in *Salmonella* phage susceptibility in laboratory cultures has been investigated by other authors (Adler et al., 2021; Barron-Montenegro et al., 2022). To our knowledge, however, this is the first study that characterizes the emergence of mechanisms involved in reduced phage-susceptibility in food biocontrol.

In the PT scenario, a low frequency of variants with reduced UAB_Phi78-susceptibility was detected. Contrary to the two previous scenarios, these variants emerged in both the untreated and the phage-treated groups but with higher frequency in the former (9.7%) than in the latter (3.3%) (**Table 3**). This low frequency of emergence of reduced phage-susceptibility variants, might be due to the synergistic effect of a significant decrease of the *Salmonella* population caused mainly by the phage cocktail and the concomitant control exerted by the host immune system as has been suggested (Salazar et al., 2021; Gaborieau and Debarbieux, 2023). Furthermore, these variants were detected in 10 (41.7%) and 3 (12.5%) of the 24 broilers of the untreated and treated groups, respectively, but with different isolation frequencies per animal. These results differ from other studies in which the reduced phage-susceptibility variants were collected mainly from treated animals (Maura and Debarbieux, 2012; Oechslin, 2018; McCallin and Oechslin, 2019; Salazar et al., 2021; Gaborieau and Debarbieux, 2023). Our findings indicated that the processes involved in the emergence of these variants in our PT experiments could be different from those taking place in previous studies and in the above described in LAB and FOOD scenarios, in which insensitive variants were identified only in treated samples.

Most of the variants with reduced UAB_Phi78-susceptibility belonging to control animals (n=62/70) also showed reduced susceptibility to the other two phages of the cocktail, while the remainder (n=8/70) displayed reduced-susceptibility to UAB_Phi87 but were susceptible to UAB_Phi20. This last pattern was also detected in all the variants (n=21) with reduced UAB_Phi78-susceptibility isolated from the treated group. The LPS electrophoretic profiles of the sixteen variants selected for analysis (**Table 6**) were not significantly different from that of the parental strain, suggesting that the observed reduced phage susceptibility might be caused by interference mechanisms with the lytic cycle of the phages and not by changes in the phage receptors. In agreement with this, the *K* values of UAB_Phi78 and UAB_Phi20 phages determined for seven of these variants (**Table 7**) confirmed the receptors’ functionality. In this regard, EOP and ECOI values were zero for UAB_Phi20 and UAB_Phi78 phages in IC3, IC5, and IC10 variants (**Table 7**), and the slight decrease of their viability without complete lysis (**Supplementary Figure S1**) suggested an abortive infection behavior (Hyman and Abedon, 2010). Likewise, the low EOP and ECOI values (lower than 1) and a high decrease of burst size exhibited by UAB_Phi20 and UAB_Phi78 phages when infecting IC6, IC8, IT2, and IT3 variants (**Table 7**) also point to this mechanism. Results for EOP and ECOI of UAB_Phi20 phage when infecting these last variants indicated that, although the infection was inefficient, it was enough to detect plaques in spot test assays (**Table 6**), which suggest that this type of assay should only be considered as a first screening tool for phage host-range and that its results must be corroborated by other methods.

The variants with reduced susceptibility to UAB_Phi87 phage deserve special mention since the phage showed inefficient adsorption and EOP values of 0. We speculate that this is caused by the involvement of phage interference in adsorption and perhaps in other processes of the phage multiplicative cycle, because no mutations were found in genes involved in LPS biosynthesis.

The comparison of whole-genome sequencing data from these variants (**Table 6**) and ATCC14028 Rif^R^ parental strain did not reveal changes in chromosomal and pSLT plasmid genes linked to previously described phage interference mechanisms. However, the detailed study of unmapped Illumina reads revealed the presence of large IncI1α plasmids in all variants and of other smaller plasmids from different incompatibility groups in some of these variants (**Table 8**). We believe that some of the ATCC14028 Rif^R^ parental bacterial cells, used to colonize the digestive tract of broilers, acquired these plasmids by lateral transfer from the broilers’ commensal gut microbiota. We suspected that the pUA1135, pUA1136, and pUA1139 plasmids contained in, respectively, IC5, IC6, and IT2 variants must encode the interference mechanisms against UAB_Phi78, UAB_Phi20 and UAB_Phi87 phages because no other exogenous plasmids were detected in these three variants. We confirmed this hypothesis through conjugation experiments, detection of plasmids on transconjugants by PCR, and curing the plasmids. EOP and ECOI values of UAB_Phi78 and UAB_Phi20 phages in transconjugants were very similar to those in donor variants (**Table 9**). Again, the data from these studies for UAB_Phi87 were inconclusive, displaying EOP values close to 0.1 (**Table 9**) and plaques with a smaller size than recipient strain *S*. Typhimurium LB5000. In this case, an EOP equal to zero would be expected since UAB_Phi87 does not present variations of adsorption on *S*. Typhimurium LB5000 and this strain is sensitive to this phage. Removing the plasmids further confirmed their implication in UAB_Phi20 and UAB_Phi78 interference, with cured strains recovering susceptibility to both phages. However, plasmid removal did not give rise to UAB_Phi87-sensitive variants like the parental strain because EOP values around 0.1 were obtained and plaque size was smaller (data not shown). Plasmid conjugation and removal results for UAB_Phi87 indicate that both a lower adsorption and interference in the multiplicative cycle of the phage could be involved in the reduced susceptibility of the variants to this phage, as discussed above. Although the role of the IncI1α plasmids in the reduced UAB_Phi87-susceptibility is clear, more studies will be required to elucidate in depth what specific processes are involved.

IncI1α plasmids are common in *Enterobacteriaceae*, and their conjugative transfer in *S*. Typhimurium causes a change of phage type to the recipient strain that has been documented *in vitro* (Hughes and Meynell, 1977; Hiley et al., 2021) and that is attributed to plasmid regions encoding phage growth inhibition systems (Furuichi et al., 1984; Sampei et al., 2010; Hiley et al., 2021). Our results indicate that this biological phenomenon could be taking place in the intestinal tract of broilers and, under certain conditions, could be the main cause of the emergence of bacterial variants with reduced susceptibility to phages in PT due to interference mechanisms encoded in the acquired plasmids. Furthermore, although receptor mutation could happen, the lateral transfer frequency in this case is most likely higher than the mutation events involved in phage resistance, because we did not detect any variants with mutations in phage receptors in PT. For the observed phenomenon to occur, the target bacterium must be at high concentration, and some of the bacteria from the environmental microbiota must harbor plasmids encoding genes involved in phage interference mechanisms and with high mating frequencies. Moreover, it is not necessary to have the phage cocktail’s selective pressure for these plasmids to be transmitted, because we isolated variants with plasmid-mediated reduced susceptibility from the untreated group of broilers.

In conclusion, and contrary to what has been previously proposed (Salazar et al., 2021; Gaborieau and Debarbieux, 2023), our findings highlight how *in vitro* assays might not anticipate the dynamics of *in vivo* reduced phage-susceptibility. This puts into question the exclusive use of *in vitro* studies to foresee bacterial phage interference mechanisms aimed for use in some kinds of therapy. Moreover, they reveal how the acquisition of plasmids in an *in vivo* scenario with a high and diverse microbiota content, such as the digestive tract, compared to FOOD and LAB scenarios resulted in bacterial reduced phage susceptibility, which to our knowledge is the first study that described this. Furthermore, this plasmid acquisition could have a larger impact than the emergence of resistance through receptor mutations. However, it is very important to highlight that the likelihood of these events does not hinder the efficacy of oral phage therapy, and our results underscore the potential of its application by using phage cocktails containing bacteriophages that recognize different receptors in the target host. Further studies are necessary to determine the plasmid regions responsible for the observed phage interference and which processes of the multiplicative cycle of the phages are affected. Finally, additional studies will be required to ascertain if the selective pressure caused by antibiotics could also play a role in the spread of anti-phage defense mechanisms and how this can influence the success of phage therapy.

## Data availability statement

The datasets presented in this study can be found in online repositories. The names of the repository/repositories and accession number(s) can be found below: https://www.ncbi.nlm.nih.gov/bioproject/PRJNA746119, https://www.ncbi.nlm.nih.gov/nuccore/MW590592, https://www.ncbi.nlm.nih.gov/nuccore/MW655523, https://www.ncbi.nlm.nih.gov/nuccore/MW65552, https://www.ncbi.nlm.nih.gov/nuccore/MW655526, https://www.ncbi.nlm.nih.gov/nuccore/MW655528, https://www.ncbi.nlm.nih.gov/nuccore/MW655529, https://www.ncbi.nlm.nih.gov/nuccore/MW655531, https://www.ncbi.nlm.nih.gov/nuccore/MW655532.

## Author contributions

JL-P: Investigation, Writing – review & editing. JO: Investigation, Writing – review & editing. MS-O: Formal Analysis, Investigation, Writing – review & editing. IE: Formal Analysis, Writing – review & editing. PC: Project administration, Supervision, Writing – original draft, Writing – review & editing. ML: Conceptualization, Project administration, Supervision, Writing – original draft, Writing – review & editing.

## References

[B1] Abuladze T. Li M. Menetrez M. Y. Dean T. Senecal A. Sulakvelidze A. (2008). Bacteriophages reduce experimental contamination of hard surfaces, tomato, spinach, broccoli, and ground beef by *Escherichia coli* O157:H7. Appl. Environ. Microbiol. 74 (20), 6230–6238. doi: 10.1128/AEM.01465-08 18723643 PMC2570303

[B2] Adler B. A. Kazako A. E. Zhong C. Liu H. Kutter E. Lui L. M.. (2021). The genetic basis of phage susceptibility, cross-resistance and host-range in *Salmonella* . Microbiology 167 (12), 1126. doi: 10.1099/mic.0.001126 PMC8744999 34910616

[B3] Altschul S. F. Madden T. L. Schäffer A. A. Zhang J. Zhang Z. Miller W.. (1997). Gapped BLAST and PSI-BLAST: a new generation of protein database search programs. Nucleic Acids Res. 25 (17), 3389–3402. doi: 10.1093/nar/25.17.3389 9254694 PMC146917

[B4] Bai J. Jeon B. Ryu S. (2019). Effective inhibition of *Salmonella* Typhimurium in fresh produce by a phage cocktail targeting multiple host receptors. Food Microbiol. 77, 52–60. doi: 10.1016/j.fm.2018.08.011 30297056

[B5] Bankevich A. Nurk S. Antipov D. Gurevich A. A. Dvorkin M. Kulikov A. S.. (2012). SPAdes: a new genome assembly algorithm and its applications to single-cell sequencing. J. Comput. Biol. 19 (5), 455–477. doi: 10.1089/cmb.2012.0021 22506599 PMC3342519

[B6] Bardina C. Spricigo D. A. Cortés P. Llagostera M. (2012). Significance of the bacteriophage treatment schedule in reducing *Salmonella* colonization of poultry. Appl. Environ. Microbiol. 78, 6600–6607. doi: 10.1128/AEM.01257-12 22773654 PMC3426709

[B7] Bardina C. Colom J. Spricigo D. A. Otero J. Sánchez-Osuna M. Cortés P.. (2016). Genomics of three new bacteriophages useful in the biocontrol of *Salmonella.* Front. Microbiol 7. doi: 10.3389/fmicb.2016.00545 PMC4837284 27148229

[B8] Barron-Montenegro R. Rivera D. Serrano M. J. García R. Álvarez D. M. Benavides J. (2022). Long-term interactions of *Salmonella* Enteritidis with a lytic phage for 21 days in high nutrients media. Front. Cell. Infect. Microbiol. 12. doi: 10.3389/fcimb.2022.897171 PMC9196899 35711664

[B9] Bertozzi Silva J. Storms Z. Sauvageau D. (2016). Host receptors for bacteriophage adsorption. FEMS Microbiol. Lett. 363 (4), fnw002. doi: 10.1093/femsle/fnw002 26755501

[B10] Bondy-Denomy J. Qian J. Westra E. R. Buckling A. Guttman D. S. Davidson A. R.. (2016). Prophages mediate defense against phage infection through diverse mechanisms. ISME J. 10 (12), 2854–2866. doi: 10.1038/ismej.2016.79 27258950 PMC5148200

[B11] Buendía-Clavería A. Moussaid A. Ollero F. Vinardell J. Torres A. Moreno J.. (2003). A *purL* mutant of *Sinorhizobium fredii* HH103 is symbiotically defective and altered in its lipopolysaccharide. Microbiology 149, 1807–1818. doi: 10.1099/mic.0.26099-0 12855732

[B12] Carattoli A. Zankari E. García-Fernández A. Voldby Larsen M. Lund O. Villa L.. (2014). *In silico* detection and typing of plasmids using PlasmidFinder and plasmid multilocus sequence typing. Antimicrob. Agents Chemother. 58 (7), 3895–3903. doi: 10.1128/AAC.02412-14 24777092 PMC4068535

[B13] Chan R. K. Botstein D. Watanabe T. Ogata Y. (1972). Specialized transduction of tetracycline resistance by phage P22 in *Salmonella* Typhimurium. II. Properties of a high-frequency-transducing lysate. Virology 50, 883–898. doi: 10.1016/0042-6822(72)90442-4 4565618

[B14] Chaveroche M. K. Ghigo J. M. d'Enfert C. (2000). A rapid method for efficient gene replacement in the filamentous fungus *Aspergillus nidulans* . Nucleic Acids Res. 28 (22), E97. doi: 10.1093/nar/28.22.e97 11071951 PMC113889

[B15] Chen W. Zhang Y. Zhang Y. Pi Y. Gu T. Song L.. (2018). CRISPR/Cas9-based genome editing in *Pseudomonas aeruginosa* and cytidine deaminase-mediated base editing in *Pseudomonas* species. iScience 6, 222–231. doi: 10.1016/j.isci.2018.07.024 30240613 PMC6137401

[B16] Colom J. Cano-Sarabia M. Otero J. Cortés P. Maspoch D. Llagostera M. (2015). Liposome-encapsulated bacteriophages for enhanced oral phage therapy against Salmonella spp. Appl. Environ. Microbiol. 81 (14), 4841–4849. doi: 10.1128/AEM.00812-15 25956778 PMC4551199

[B17] Colom J. Cano-Sarabia M. Otero J. Aríñez-Soriano J. Cortés P. Maspoch D.. (2017). Microencapsulation with alginate/CaCO_3_: A strategy for improved phage therapy. Sci. Rep. 7, 41441. doi: 10.1038/srep41441 28120922 PMC5264180

[B18] Darling A. E. Mau B. Perna N. T. (2010). Progressive Mauve: multiple genome alignment with gene gain, loss and rearrangement. PloS One 5 (6), e11147. doi: 10.1371/journal.pone.0011147 20593022 PMC2892488

[B19] Datsenko K. A. Wanner B. L. (2000). One-step inactivation of chromosomal genes in *Escherichia coli* K-12 using PCR products. Proc. Natl. Acad. Sci. U.S.A. 97 (12), 6640–6645. doi: 10.1073/pnas.120163297 10829079 PMC18686

[B20] de Jonge P. A. Nobrega F. L. Brouns S. J. J. Dutilh B. E. (2019). Molecular and evolutionary determinants of bacteriophage host range. Trends Microbiol. 27 (1), 51–63. doi: 10.1016/j.tim.2018.08.006 30181062

[B21] de Moraes M. H. Teplitski M. (2015). Fast and efficient three-step target-specific curing of a virulence plasmid in *Salmonella enterica* . AMB Express 5 (1), 139. doi: 10.1186/s13568-015-0139-y 26272479 PMC4536245

[B22] De Sordi L. Lourenço M. Debarbieux L. (2019). "I will survive": A tale of bacteriophage-bacteria coevolution in the gut. Gut Microbes 10 (1), 92–99. doi: 10.1080/19490976.2018.1474322 29913091 PMC6363074

[B23] Doron S. Melamed S. Ofir G. Leavitt A. Lopatina A. Keren M.. (2018). Systematic discovery of antiphage defense systems in the microbial pangenome. Science 359 (6379), eaar4120. doi: 10.1126/science.aar4120 29371424 PMC6387622

[B24] Dy R. L. Przybilski R. Semeijn K. Salmond G. P. C. Fineran P. C. A. (2014). Widespread bacteriophage abortive infection system functions through a type IV toxin–antitoxin mechanism. Nucleic Acids Res. 42, 4590–4605. doi: 10.1093/nar/gkt1419 24465005 PMC3985639

[B25] Egido J. E. Costa A. R. Aparicio-Maldonado C. Haas P. J. Brouns S. J. J. (2022). Mechanisms and clinical importance of bacteriophage resistance. FEMS Microbiol. Rev. 46 (1), fuab048. doi: 10.1093/femsre/fuab048 34558600 PMC8829019

[B26] European Antimicrobial Resistance Collaborators (2022). The burden of bacterial antimicrobial resistance in the WHO European region in 2019: a cross-country systematic analysis. Lancet Public Health 7 (11), e897–e913. doi: 10.1016/S2468-2667(22)00225-0 36244350 PMC9630253

[B27] Finn R. D. Coggill P. Eberhardt R. Y. Eddy S. R. Mistry J. Mitchell A. L.. (2016). The Pfam protein families database: towards a more sustainable future. Nucleic Acids Res. 44 (D1), D279–D285. doi: 10.1093/nar/gkv1344 26673716 PMC4702930

[B28] Furuichi T. Komano T. Nisioka T. (1984). Physical and genetic analyses of the Inc-I alpha plasmid R64. J. Bacteriol. 158 (3), 997–1004. doi: 10.1128/jb.158.3.997-1004.1984 6327656 PMC215541

[B29] Gaborieau B. Debarbieux L. (2023). The role of the animal host in the management of bacteriophage resistance during phage therapy. Curr. Opin. Virol. 58, 101290. doi: 10.1016/j.coviro.2022.101290 36512896

[B30] García-Quintanilla M. Ramos-Morales F. Casadesús J. (2008). Conjugal transfer of the *Salmonella enterica* virulence plasmid in the mouse intestine. J. Bacteriol. 190 (6), 1922–1927. doi: 10.1128/JB.01626-07 18178735 PMC2258861

[B31] Goldfarb T. Sberro H. Weinstock E. Cohen O. Doron S. Charpak-Amikam Y.. (2015). BREX is a novel phage resistance system widespread in microbial genomes. EMBO J. 34 (2), 169–183. doi: 10.15252/embj.201489455 25452498 PMC4337064

[B32] Gordillo Altamirano F. Barr J. (2019). Phage therapy in the postantibiotic era. Clin. Microbiol. Rev. 32 (2), e00066–e00018. doi: 10.1128/CMR.00066-18 30651225 PMC6431132

[B33] Górski A. Międzybrodzki R. Łobocka M. Głowacka-Rutkowska A. Bednarek A. Borysowski J.. (2018). Phage therapy: what have we learned? Viruses 10 (6), 288. doi: 10.3390/v10060288 29843391 PMC6024844

[B34] Guttman B. Raya R. Kutter E. (2005). “Basic phage biology,” in Bacteriophages: Biology and Application. Eds. Kutter E. Sulakvelidze A. (Boca Raton, FL: CRC Press), 29–66.

[B35] Hernandez C. A. Koskella B. (2019). Phage resistance evolution in *vitro* is not reflective of in *vivo* outcome in a plant-bacteria-phage system. Evolution 73 (12), 2461–2475. doi: 10.1111/evo.13833 31433508

[B36] Hiley L. Graham R. M. A. Jennison A. V. (2021). Characterisation of IncI1 plasmids associated with change of phage type in isolates of *Salmonella enterica* serovar Typhimurium. BMC Microbiol. 21 (1), 92. doi: 10.1186/s12866-021-02151-z 33773572 PMC8004404

[B37] Hille F. Richter H. Wong S. P. Bratovič M. Ressel S. Charpentier E. (2018). The biology of CRISPR-Cas: backward and forward. Cell 172 (6), 1239–1259. doi: 10.1016/j.cell.2017.11.032 29522745

[B38] Hitchcock P. J. Leivem L. Mäkeläm P. H. Rietschel E. T. Strittmatter W. Morrison D. C. (1986). Lipopolysaccharide nomenclature-past, present, and future. J. Bacteriol. 166 (3), 699–705. doi: 10.1128/jb.166.3.699-705.1986 2872204 PMC215174

[B39] Hong Y. Schmidt K. Marks D. Hatter S. Marshall A. Albino L.. (2016). Treatment of *Salmonella* -contaminated eggs and pork with a broad-spectrum, single bacteriophage: Assessment of efficacy and resistance development. Foodborne Pathog. Dis. 13 (12), 679–688. doi: 10.1089/fpd.2016.2172 27768383

[B40] Hughes V. Meynell G. G. (1977). The contribution of plasmid and host genes to plasmid-mediated interference with phage growth. Genet. Res. 30 (2), 179–185. doi: 10.1017/s0016672300017572 924144

[B41] Hyman P. Abedon S. T. (2010). Bacteriophage host range and bacterial resistance. Adv. Appl. Microbiol. 70, 217–248. doi: 10.1016/S0065-2164(10)70007-1 20359459

[B42] Isaev A. B. Musharova O. S. Severinov K. V. (2021a). Microbial arsenal of antiviral defenses. Part I. Biochem. (Mosc) 86 (3), 319–337. doi: 10.1134/S0006297921030081 33838632

[B43] Isaev A. B. Musharova O. S. Severinov K. V. (2021b). Microbial arsenal of antiviral defenses. Part II. Biochem. (Mosc) 86 (4), 449–470. doi: 10.1134/S0006297921040064 33941066

[B44] Jones G. W. Rabert D. K. Svinarich D. M. Whitfield H. J. (1982). Association of adhesive, invasive, and virulent phenotypes of *Salmonella* Typhimurium with autonomous 60-megadalton plasmids. Infect. Immun. 38 (2), 476–486. doi: 10.1128/iai.38.2.476-486.1982 6128304 PMC347764

[B45] Khan Mirzaei M. Nilsson A. S. (2015). Isolation of phages for phage therapy: a comparison of spot tests and efficiency of plating analyses for determination of host range and efficacy. PloS One 10 (3), e0118557. doi: 10.1371/journal.pone.0118557 25761060 PMC4356574

[B46] Kropinski A. M. (2009). Measurement of the rate of attachment of bacteriophage to cells. Methods Mol. Biol. 501, 151–155. doi: 10.1007/978-1-60327-164-6_15 19066819

[B47] Kropinski A. M. (2018). Practical advice on the one-step growth curve. Methods Mol. Biol. 1681, 41–47. doi: 10.1007/978-1-4939-7343-9_3 29134585

[B48] Kutter E. (2009). Phage host range and efficiency of plating. Methods Mol. Biol. 501, 141–149. doi: 10.1007/978-1-60327-164-6_14 19066818

[B49] Levin B. R. Bull J. J. (2004). Population and evolutionary dynamics of phage therapy. Nat. Rev. Microbiol. 2 (2), 166–173. doi: 10.1038/nrmicro822 15040264

[B50] Lopatina A. Tal N. Sorek R. (2020). Abortive infection: bacterial suicide as an antiviral immune strategy. Annu. Rev. Virol. 7 (1), 371–384. doi: 10.1146/annurev-virology-011620-040628 32559405

[B51] Makarova K. S. Wolf Y. I. van der Oost J. Koonin E. V. (2009). Prokaryotic homologs of Argonaute proteins are predicted to function as key components of a novel system of defense against mobile genetic elements. Biol. Direct. 25 (4), 29. doi: 10.1186/1745-6150-4-29 PMC2743648 19706170

[B52] Maura D. Debarbieux L. (2012). On the interactions between virulent bacteriophages and bacteria in the gut. Bacteriophage. 1;2 4), 229–233. doi: 10.4161/bact.23557 PMC3594211 23739386

[B53] Mayola A. Irazoki O. Martínez I. A. Petrov D. Menolascina F. Stocker R.. (2014). RecA protein plays a role in the chemotactic response and chemoreceptor clustering of *Salmonella enterica* . PloS One 9 (8), e105578. doi: 10.1371/journal.pone.0105578 25147953 PMC4141790

[B54] McCallin S. Oechslin F. (2019). “Bacterial resistance to phage and its impact on clinical therapy,” in Phage Therapy: A Practical Approach. Eds. Górski A. Międzybrodzki R. Borysowski J. (© Springer Nature Switzerland AG 2019), 59–91.

[B55] Millman A. Bernheim A. Stokar-Avihail A. Fedorenko T. Voichek M. Leavitt A.. (2020). Bacterial retrons function in anti-phage defense. Cell 183 (6), 1551–1561.e12. doi: 10.1016/j.cell.2020.09.065 33157039

[B56] Millman A. Melamed S. Leavitt A. Doron S. Bernheim A. Hör J.. (2022). An expanding arsenal of immune systems that protect bacteria from phages. Cell Host Microbe 30 (11), 1556–1569.e5. doi: 10.1016/j.chom.2022.09.017 36302390

[B57] Moineau S. Durmaz E. Pandian S. Klaenhammer T. R. (1993). Differentiation of two abortive mechanisms by using monoclonal antibodies directed toward lactococcal bacteriophage capsid proteins. Appl. Environ. Microbiol. 59 (1), 208–212. doi: 10.1128/aem.59.1.208-212.1993 16348844 PMC202079

[B58] Oechslin F. Piccardi P. Mancini S. Gabard J. Moreillon P. Entenza J. M.. (2017). Synergistic interaction between phage therapy and antibiotics clears *Pseudomonas aeruginosa* infection in endocarditis and reduces virulence. J. Infect. Dis. 215 (5), 703–712. doi: 10.1093/infdis/jiw632 28007922 PMC5388299

[B59] Oechslin F. (2018). Resistance development to bacteriophages occurring during bacteriophage therapy. Viruses 10 (7), 351. doi: 10.3390/v10070351 29966329 PMC6070868

[B60] Ofir G. Melamed S. Sberro H. Mukamel Z. Silverman S. Yaakov G.. (2018). DISARM is a widespread bacterial defence system with broad anti-phage activities. Nat. Microbiol. 3 (1), 90–98. doi: 10.1038/s41564-017-0051-0 29085076 PMC5739279

[B61] Pedruzzi I. Rivoire C. Auchincloss A. H. Coudert E. Keller G. de Castro E.. (2015). HAMAP in 2015: updates to the protein family classification and annotation system. Nucleic Acids Res. 43, D1064–D1070. doi: 10.1093/nar/gku1002 25348399 PMC4383873

[B62] Pereira C. Moreirinha C. Lewicka M. Almeida P. Clemente C. Cunha Â.. (2016). Bacteriophages with potential to inactivate *Salmonella* Typhimurium: use of single phage suspensions and phage cocktails. Virus Res. 220, 179–192. doi: 10.1016/j.virusres.2016.04.020 27126773

[B63] Rousset F. Depardieu F. Miele S. Dowding J. Laval A. L. Lieberman E.. (2022). Phages and their satellites encode hotspots of antiviral systems. Cell. Host. Microbe 30 (5), 740–753.e5. doi: 10.1016/j.chom.2022.02.018 35316646 PMC9122126

[B64] Salazar K. C. Ma L. Green S. I. Zulk J. J. Trautner B. W. Ramig R. F.. (2021). Antiviral resistance and phage counter adaptation to antibiotic-resistant extraintestinal pathogenic *Escherichia coli* . mBio 12 (2), e00211–e00221. doi: 10.1128/mBio.00211-21 33906920 PMC8092219

[B65] Sampei G. Furuya N. Tachibana K. Saitou Y. Suzuki T. Mizobuchi K.. (2010). Complete genome sequence of the incompatibility group I1 plasmid R64. Plasmid 64 (2), 92–103. doi: 10.1016/j.plasmid.2010.05.005 20594994

[B66] Sánchez-Osuna M. Cortés P. Barbé J. Erill I. (2019). Origin of the mobile di-hydro-pteroate synthase gene determining sulfonamide resistance in clinical isolates. Front. Microbiol. 9. doi: 10.3389/fmicb.2018.03332 PMC6335563 30687297

[B67] Schnaitman C. A. Klena J. D. (1993). Genetics of lipopolysaccharide biosynthesis in enteric bacteria. Microbiol. Rev. 57 (3), 655–682. doi: 10.1128/mr.57.3.655-682.1993 7504166 PMC372930

[B68] Schooley R. T. Biswas B. Gill J. J. Hernandez-Morales A. Lancaster J. Lessor L.. (2017). Development and use of personalized bacteriophage-based therapeutic cocktails to treat a patient with a disseminated resistant *Acinetobacter baumannii* infection. Antimicrob. Agents Chemother. 61 (10), e00954–e00917. doi: 10.1128/AAC.00954-17 28807909 PMC5610518

[B69] Seemann T. (2014). Prokka: rapid prokaryotic genome annotation. Bioinformatics 30 (14), 2068–2069. doi: 10.1093/bioinformatics/btu153 24642063

[B70] Takamatsu D. Osaki M. Sekizaki T. (2001). Thermosensitive suicide vectors for gene replacement in *Streptococcus suis* . Plasmid 46 (2), 140–148. doi: 10.1006/plas.2001.1532 11591139

[B71] Tatusov R. L. Galperin M. Y. Natale D. A. Koonin E. V. (2000). The COG database: a tool for genome-scale analysis of protein functions and evolution. Nucleic Acids Res. 28 (1), 33–36. doi: 10.1093/nar/28.1.33 10592175 PMC102395

[B72] Tock M. R. Dryden D. T. F. (2005). The biology of restriction and anti-restriction. Curr. Opin. Microbiol. 8, 466–472. doi: 10.1016/j.mib.2005.06.003 15979932

[B73] Tomat D. Casabonne C. Aquili V. Balagué C. Quiberoni A. (2018). Evaluation of a novel cocktail of six lytic bacteriophages against Shiga toxin-producing *Escherichia coli* in broth, milk and meat. Food Microbiol. 76, 434–442. doi: 10.1016/j.fm.2018.07.006 30166171

[B74] Tu J. Park T. Morado D. R. Hughes K. T. Molineux I. J. Liu J. (2017). Dual host specificity of phage SP6 is facilitated by tailspike rotation. Virology 507, 206–215. doi: 10.1016/j.virol.2017.04.017 28456019 PMC5503682

[B75] Vielva L. de Toro M. Lanza V. F. de la Cruz F. (2017). PLACNETw: a web-based tool for plasmid reconstruction from bacterial genomes. Bioinformatics 33 (23), 3796–3798. doi: 10.1093/bioinformatics/btx462 29036591

[B76] von Wintersdorff C. J. Penders J. van Niekerk J. M. Mills N. D. Majumder. S. van Alphen L. B.. (2016). Dissemination of antimicrobial resistance in microbial ecosystems through horizontal gene transfer. Front. Microbiol. 7. doi: 10.3389/fmicb.2016.00173 PMC4759269 26925045

[B77] Wright R. C. T. Friman V. P. Smith M. C. M. Brockhurst M. A. (2019). Resistance evolution against phage combinations depends on the timing and order of exposure. mBio 10 (5), e01652–e01619. doi: 10.1128/mBio.01652-19 31551330 PMC6759759

[B78] Zerbino D. R. (2010). Using the Velvet *de novo* assembler for short-read sequencing technologies. Curr. Protoc. Bioinf. 31 (11.5), 1-12. doi: 10.1002/0471250953.bi1105s31 PMC2952100 20836074

