# Supplementary material for: Impact of mutagenesis and lateral gene transfer processes in bacterial susceptibility to phage in food biocontrol and phage therapy
Source: Front Cell Infect Microbiol. 2023 Sep 28;13:1266685. doi: 10.3389/fcimb.2023.1266685 (PMC10569123; doi:10.3389/fcimb.2023.1266685)
Supplement: Supplementary file 1 [file DataSheet_1.docx]

Supplementary Material

**Supplementary Figure 1**. Growth kinetics, measured as absorbance (A_550_nm) (●) and viable count (▲) of cultures of the parental strain ATCC 14028Rif^R^**(A)** and the variants IC10 **(B)** and IT2 **(C)** as examples of those isolated from PT, non-infected (solid line) and infected (dot line) with UAB_Phi78 bacteriophage. Arrow indicates the time of phage infection of cultures. The standard deviation of at least three experiments is shown.
