# Supplementary material for: Impact of mutagenesis and lateral gene transfer processes in bacterial susceptibility to phage in food biocontrol and phage therapy
Source: Front Cell Infect Microbiol. 2023 Sep 28;13:1266685. doi: 10.3389/fcimb.2023.1266685 (PMC10569123; doi:10.3389/fcimb.2023.1266685)
Supplement: Supplementary file 2 [file DataSheet_2.docx]

Supplementary Material

**Supplementary Figure 2**. LPS electrophoretic profiles of the sixteen ATCC 14028Rif^R^ variants isolated from PT, The LPS electrophoretic profile of the ATCC 14028 Rif^R^ parental strain is also shown as control.
