# Supplementary figures and images for: Impact of mutagenesis and lateral gene transfer processes in bacterial susceptibility to phage in food biocontrol and phage therapy
Source: Front Cell Infect Microbiol. 2023 Sep 28;13:1266685. doi: 10.3389/fcimb.2023.1266685 (PMC10569123; doi:10.3389/fcimb.2023.1266685)

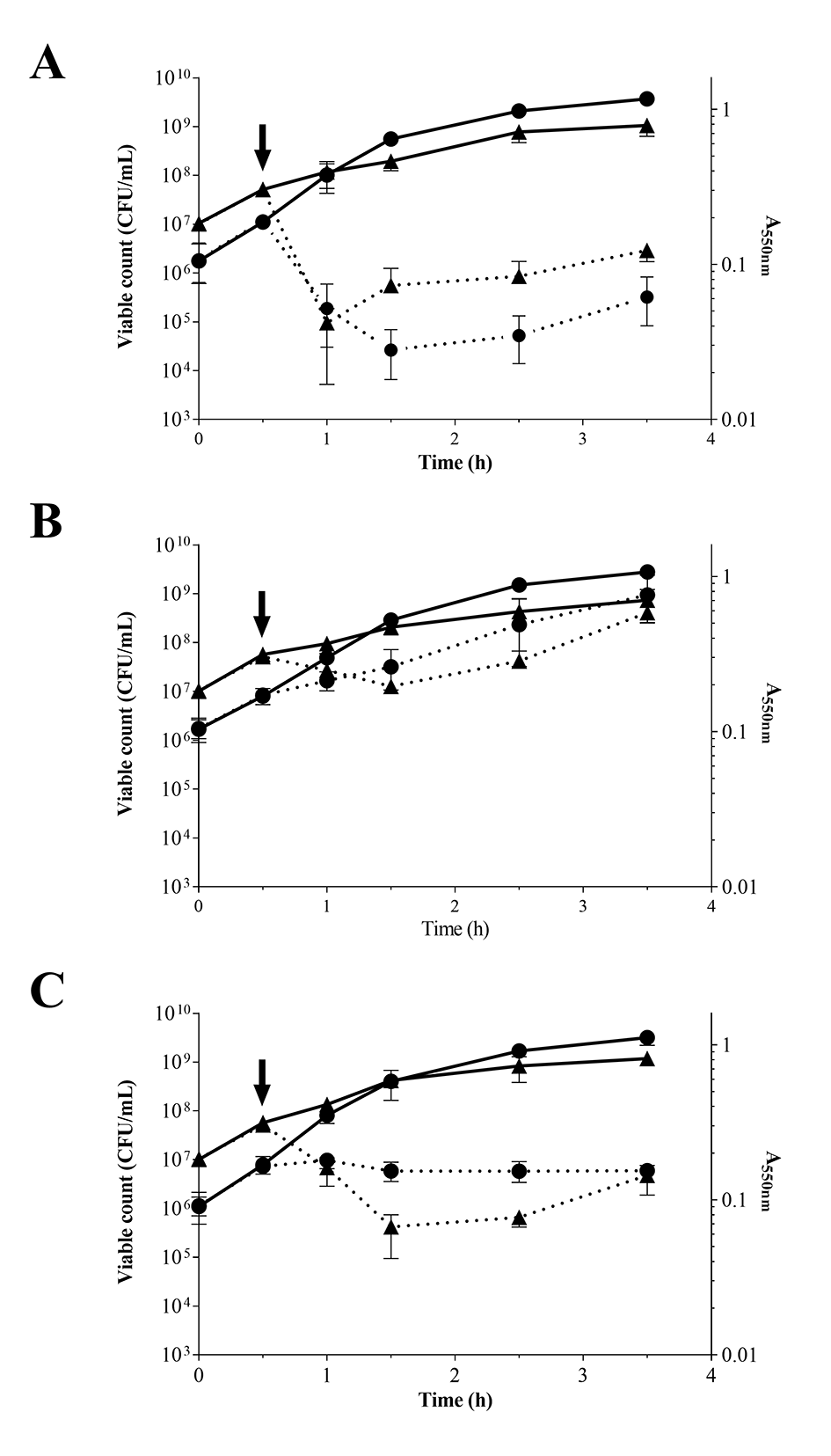

Supplement: Supplementary file 3 [file Image_1.tif]

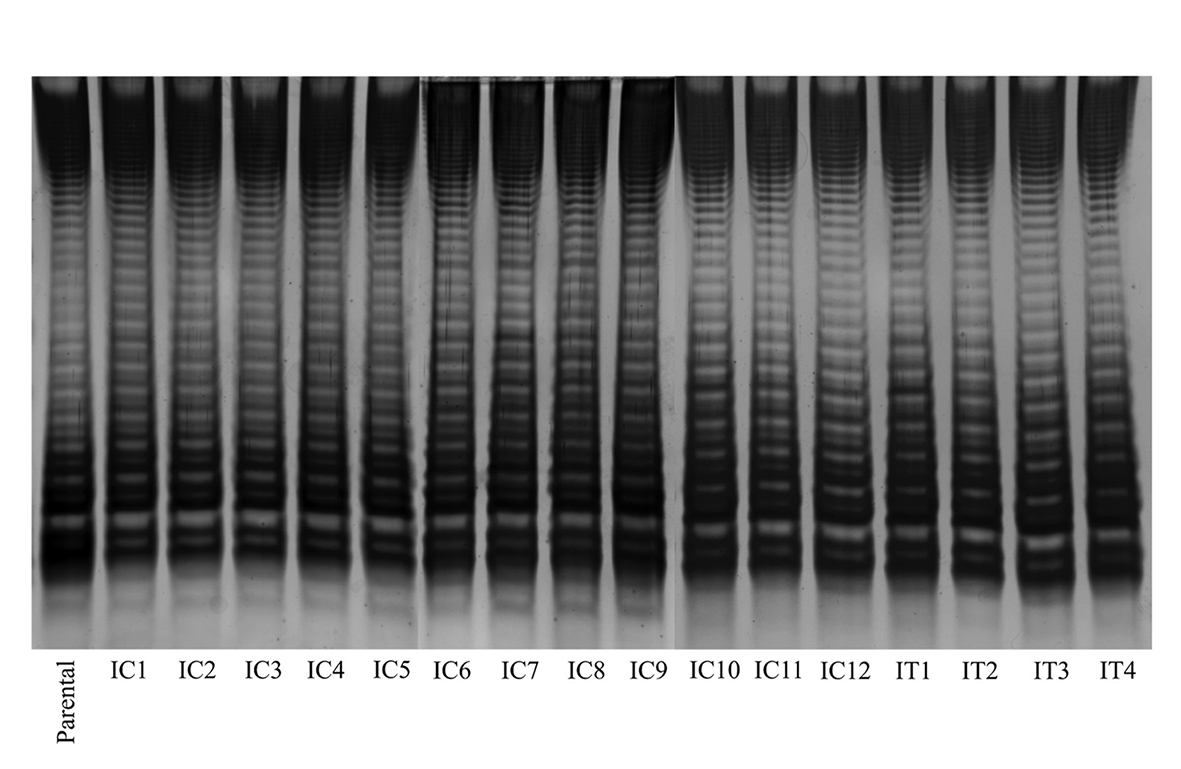

Supplement: Supplementary file 4 [file Image_2.tif]
