# Supplementary material for: Impact of mutagenesis and lateral gene transfer processes in bacterial susceptibility to phage in food biocontrol and phage therapy
Source: Front Cell Infect Microbiol. 2023 Sep 28;13:1266685. doi: 10.3389/fcimb.2023.1266685 (PMC10569123; doi:10.3389/fcimb.2023.1266685)
Supplement: Supplementary file 5 [file Table_1.docx]

Supplementary Material

**Supplementary Table 1**

**Table S1**. Strains and plasmids used in this study.

|  | **Relevant characteristics** | **Source or reference** |
| --- | --- | --- |
| **Strains** |  |  |
| ATCC 14028 Rif^R^ | *S.* Typhimurium *rfa* (+); smooth LPS*;* Rif^R^ | Universitat Autònoma de Barcelona |
| LB5000 | *S.* Typhimurium *metA22 metE551 trpC2 ilv-452 H1-b metA22 metE551 trpC2 ilv-452 H1-b H2-e,n,x* (cured of Fels 2) *fla-66 rpsL120 xyl-404 leu hsdL6 hsdSA29 hsdSB* | University of Calgary |
| DH5α | *E. coli* *supE4 ΔlacU169* (φ80 ΔlacZ ΔM15) *hsdR17, recA1, endA1, gyrA96, thi-1, relA1* | Clontech |
| TA1537 | *S.* Typhimurium *hisC3076 rfa uvrB*; deep rough LPS | Dr. Bruce Ames (University of California, Berkeley) |
| SL3770 | *S.* Typhimurium *rfa* (+); smooth LPS | *Salmonella* Genectic Stock Center (University of Calgary) |
| SL4807 | *S.* Typhimurium rfaB707; mixture of Rc and smooth |  |
| SL428 | *S.* Typhimurium *rfc-*458; Ra with 1 side chain unit |  |
| SL3749 | *S.* Typhimurium *rfaL446;* Ra |  |
| SL733 | *S.* Typhimurium *rfaK953;* Rb1 |  |
| SL3750 | *S.* Typhimurium *rfaJ417;* Rb2 |  |
| SL1306 | *S.* Typhimurium *galE503;* Rc |  |
| SL3769 | *S.* Typhimurium *rfaG471;* Rd1 |  |
| SL3789 | *S.* Typhimurium *rfaF511;* Rd2 |  |
| SL1102 | *S.* Typhimurium *rfaE543;* Re |  |
| **Plasmids** |  |  |
| pKD4 | Amp^R^ Km^R^ | Datsenko and Warner, 2000 |
| pKD46 | Vector containing the λ Red recombinase system, Amp^R^, thermosensitive |  |
| pCP20 | Vector carrying FLP system, OriV, thermosensitive, Amp^R^ |  |
| pKOBEG | Vector containing the λ Red recombinase system, Cm^R^, thermosensitive | Generous gift of Prof. I. Lasa; Chaveroche et al., 2000 |
| pUA1108 | pGEX 4T-1 derivative plasmid carrying the *Ptac* promoter and the *lacIq* gene; Amp^R^ | Mayolla et al., 2014 |
| pCasPA | Vector codifying Cas9 nuclease and λ-Red recombination system, Tet^R^, sucrose counter-selectable, Amp^R^ | Generous gift of Prof. Quanjiang Ji (ShanghaiTech). Chen et al., 2018. |
| pACRISPR | Vector codifying sgRNA for targeting a specific sequence, Amp^R^ |  |
| pSET4s | Replication function of pG + host3 and pUC19, *lacZ*’ Spt^R^ | Generous gift of Dr. T. Sekizaki. Takamatsu *et a*l., 2001 |
| pUA1148 | pACRISPR derivative; Spt^R^ | This study |
| pUA1148+gRNA48 | pUA1148 derivative for pUA1135 and pUA1136 elimination |  |
| pUA1165 | pKD46 plasmid without λ-Red system genes and with I-*Sce*I endonuclease under control of tetracycline inducible promoter (P_tetA_), thermosensitive, Amp^R^ |  |

* Ra, Rb1, Rb2, Rb3, Rc, Rd1, and Rd2 are rough chemotypes. Re is a deep rough chemotype (Hitchcock *et al*., 1986). LPS, lipopolysaccharide. Amp, ampicillin; Cm, chloramphenicol; Km, kanamycin; Rif, rifampicin; Spt, spectinomycin; Tet, tetracycline. ^R^ resistant.
