# Supplementary material for: Impact of mutagenesis and lateral gene transfer processes in bacterial susceptibility to phage in food biocontrol and phage therapy
Source: Front Cell Infect Microbiol. 2023 Sep 28;13:1266685. doi: 10.3389/fcimb.2023.1266685 (PMC10569123; doi:10.3389/fcimb.2023.1266685)
Supplement: Supplementary file 6 [file Table_2.docx]

Supplementary Material

**Supplementary Table 2**

**Table S2**. Oligonucleotides used in this work.

| **Name** | **Sequence (5’-3’)** | **Application** | **Reference** |
| --- | --- | --- | --- |
| rfc_F | GTTGGTAATGATGGCTCA | PCR and sequencing of *rfc* | This study |
| rfc_rv | CACCTTATTTGCCTGATG |  |  |
| rfc_int_F | GACTGCTATAACAGCAAAC | Sequencing of *rfc* |  |
| rfc_int_rv | GTTTGCTGTTATAGCAGTC |  |  |
| HiFirfc_F | TTCACACAGGAAACAGTACAATGCTTATAATTTCATACATTGC | PCR of *rfc* for HIFI cloning on pUA1108 |  |
| HiFirfc_Rv | TCGACCCGGGGAATTCCGGGTTATTTATTGTTTCTTAGTAAAACGAATC |  |  |
| HIFIrfaJ_F | TTCACACAGGAAACAGTACAATGGATTCATTTCCTGAGATAGAAATAG | PCR of *rfaJ* for HIFI cloning on pUA1108 |  |
| HIFIrfaJ_R | TCGACCCGGGGAATTCCGGGTTATTTGTGGAAAAGTTTACGATAAAG |  |  |
| Rep1_fw | CTCCTGTTCCTGCCTTTC | Detection of plasmid pUA1144 |  |
| Rep1_rv | CTTTCCGGGTTGGACTC |  |  |
| Rep2_fw | GTGAGCACTCCACCAAAC | Detection of plasmids pUA1135, pUA1136, and pUA1139 |  |
| Rep2_rv | GACGCCCAGCATCTG |  |  |
| Abi5_fw | GCAGATATTATTCGGATGCAAC | Detection of plasmid pUA1141 |  |
| Abi5_rv | CTGTTAAATAATCGGGTGTGG |  |  |
| kikA_fw | GTGGTTTTACCAGCCTTG | Detection of plasmid pUA1138 |  |
| kikA_rv | CTTTAACCTTGCCGAACTTAC |  |  |
| Rep3_fw | GCTGCGATTGACTGGAG | Detection of plasmid pUA1142 |  |
| Rep3_rv | CAATAGCCCGACATTCAG |  |  |
| Rep4_fw | ctgcgctgaatatggcg | Detection of plasmid pUA1145 |  |
| Rep4_rv | CCTGTTTCACTTTCGGTC |  |  |

**Table S2.** (continued)

| **Name** |  | **Sequence (5’-3’)** | **Application** | **Reference** |
| --- | --- | --- | --- | --- |
| P4_Km_P1^a^ |  | GGAAACCGCCCGTTTCTTTTCCGACAAAGGATGTCGCCGTGCTCCTCTTTTT*gtgtaggctggagctgcttc* | PCR of Km gene from pKD4 plasmid with homology regions of the intergenic region of the plasmid pUA1139 of the strains IT2 and IT3 | This study |
| P4_Km_P2^a^ |  | GCCGGACGCGGGACACTGACCACAGGCAGCGTTTATGCCGCGCCGCGTCAGC*atgggaattagccatggtcc* |  |  |
| P4km_extf |  | Cattatggcggatgaagtatg | PCR and sequencing to check the Km insertion into the intergenic region of the strains IT2 and IT3 |  |
| P4km_extr |  | Ctggcgtacactgtcac |  |  |
| pACRISPR_fwd |  | CTGTCAGACCAAGTTTAC | Inverse PCR to amplify pACRISPR plasmid (without *bla* gene) for HIFI cloning |  |
| pACRISPR_rev |  | ACTCTTCCTTTTTCAATATTATTG |  |  |
| Spc_fwd |  | AATATTGAAAAAGGAAGAGTGTGAGGAGGATATATTTGAATAC | PCR to amplify *spt* gene from pSET4s for HIFI cloning |  |
| Spc_rev |  | GAGTAAACTTGGTCTGACAGTTATAATTTTTTTAATCTGTTATTTAAATAGTTTATAG |  |  |
| Spc_int_fw |  | GAGGAATTACTACCTGATATTC | PCR to check the pUA1148 plasmid construction |  |
| pACRISPR_rv2 |  | CTGGTAACAGGATTAGCAG |  |  |
| gRNA48_fw ^b^ |  | GTGGGTGTTTCAGAGGAAACCCTG | Phosphorylated primers to anneal the gRNA48 spacer through Golden Gate assembly |  |
| gRNA48_rv ^b^ |  | AAACCAGGGTTTCCTCTGAAACAC |  |  |
| pACRISPR2622_rv |  | CATGGGTATGGACAGATC | PCR and sequencing of cloned gRNA48 spacer in pUA1148 |  |
| pACRISPR2431_fw |  | GTGCCAATACCAGTAGAAAC |  |  |
| *Sce*I_sec_rv |  | TCGATGTTCAGTTCGATCAG |  |  |
| pUA1139_00007_km_p1.2 ^a,c^ |  | TACAGATGCACCGATGCGCTTGCGTGGCTTTTCAGGGAGATAAAGTTATGAGTTACGCTAGGGATAACAGGGTAATATAG*taggaacttcaagatcccct* | PCR of Km gene from pKD4 plasmid with homology regions of the *pUA1139_00007* gene of the plasmid pUA1139 |  |
| pUA1139_00007_km_p2.1 ^a^ |  | TTCCGGCACAGGTGAGGCCGGAATTCGGACTAAAACGTAAACCGCGGGCC*atgggaattagccatggtcc* |  |  |
| pUA1139_00007_ext_fw |  | GCTGAGTGAAAACACCCTT | PCR and sequencing to check the Km insertion into the *pUA1139_00007* gene |  |
| pUA1139_00007_ext_rv_2 |  | GGCTGAAGGCCTTTATAGT |  |  |

**Table S2.** (continued)

| **Name** | **Sequence (5’-3’)** | **Application** | **Reference** |
| --- | --- | --- | --- |
| pKD46_inv_fw | CGAAATCCACTGAAAGCACA | Inverse PCR to amplify pKD46 plasmid without λ-Red system genes | de Moraes and Teplitski, 2015 |
| pKD46_inv_rv | AAGCTGCTTTTGAGCACCAC |  |  |
| *Sce*I_pKD46_fw | TACCAGTAGAAACAGACGAAGAATCGACCAATTCGGGTCGACTTATTATTTC | Amplification of I-*Sce*I synthetic gene and tetracycline inducible promoter | This study |
| *Sce*I_pKD46_rv | TATCAAAGGGAAAACTGTCCATACCGGAAAAAGGTTATGCTGCTTTTAAG |  |  |
| pKD46_mod_comp_fw | GACAACTTGACGGCTACA | PCR and sequencing to check the pUA1165 plasmid construction. |  |
| pKD46_mod_comp_rv | GAACAACTGTTCACCGTTACA |  |  |
| *Sce*I_sec_fw | CTGATCGAACTGAACATCGA |  |  |
| *Sce*I_sec_rv | TCGATGTTCAGTTCGATCAG |  |  |

^a^ P1 and P2 sequences, homologues to the pKD4 plasmid, are represented in lower case italics; ^b^ Regions of homology with pUA1148 are represented underlined; ^c^ I-*Sce*I restriction site is represented underlined.
