# Supplementary material for: Impact of mutagenesis and lateral gene transfer processes in bacterial susceptibility to phage in food biocontrol and phage therapy
Source: Front Cell Infect Microbiol. 2023 Sep 28;13:1266685. doi: 10.3389/fcimb.2023.1266685 (PMC10569123; doi:10.3389/fcimb.2023.1266685)
Supplement: Supplementary file 7 [file Table_3.docx]

Supplementary Material

**Supplementary Table 3**

|  |  |  |  |  | **Mutation Frequency (%)** | | | | | | | | | | | |
| --- | --- | --- | --- | --- | --- | --- | --- | --- | --- | --- | --- | --- | --- | --- | --- | --- |
| **Locus tag** | **Gene** | **Function^a^** | **CDS position** | **Codon change** | **Parental** | **CI2** | **CI3** | **HT1** | **HT5** | **IC3** | **IC5** | **IC6** | **IC8** | **IC10** | **IT2** | **IT3** |
| STM14_1424 | Prophage *RecE* | Exodeoxyribonuclease VIII | 281 | G**A**T/G**-**T | 99.7 | 99.8 | 99.8 | 99.8 | 99.8 | 99.7 | 99.7 | 99.7 | 99.7 | 99.8 | 99.8 | 98.9 |
| STM14_1478 | Side tail fiber protein | Unknown | 1875 | AA**T**/AA**A** | 99.4 | 99.2 | 99.8 | 99.2 | 99.7 | 99.4 | 99.5 | 99.4 | 99.7 | 99.4 | 99.7 | 100.0 |
| STM14_1964 | *ydcJ* | Cytoplasmic protein, unknown function | 1084 | **A**CG/**G**CG | 99.7 | 99.7 | 99.5 | 99.8 | 99.4 | 99.7 | 99.5 | 99.7 | 99.7 | 99.5 | 99.7 | 100.0 |
| STM14_2274 | *RecE-like* | Exodeoxyribonuclease VIII | 350 | G**G**C/G**A**C | 99.8 | 99.7 | 99.8 | 99.8 | 99.7 | 99.8 | 99.5 | 99.7 | 99.7 | 99.8 | 99.8 | 100.0 |
|  |  |  | 357 | AG**T**/AG**C** | 99.8 | 99.2 | 99.8 | 99.8 | 99.7 | 99.8 | 99.7 | 99.4 | 99.7 | 99.7 | 99.8 | 100.0 |
| STM14_2439 |  | Phage protein | 264 | AA**T**/AA**-** | 99.7 | 99.5 | 99.6 | 99.8 | 99.7 | 99.7 | 99.7 | 99.4 | 99.7 | 99.7 | 99.0 | 90.5 |
| STM14_2767 |  | Putative cytoplasmic protein, unknown function | 64 | **T**CG/**G**CG | 99.4 | 99.4 | 99.5 | 99.7 | 99.7 | 99.4 | 99.7 | 99.6 | 99.6 | 99.7 | 99.7 | 100.0 |
|  |  |  | 125 | T**T**T/T**A**T | 99.7 | 99.8 | 99.8 | 99.8 | 99.7 | 99.7 | 99.7 | 99.7 | 99.7 | 99.8 | 99.5 | 100.0 |
|  |  |  | 139 | **A**GC/**T**GC | 99.7 | 99.7 | 99.5 | 99.8 | 99.7 | 99.7 | 99.7 | 99.7 | 99.1 | 99.8 | 99.3 | 100.0 |
| STM14_2859 | *nuoL* | Proton-translocating NADH-quinone oxidoreductase subunit L | 617 | G**T**G/G**C**G | 99.7 | 99.7 | 99.7 | 99.8 | 99.7 | 99.7 | 99.5 | 99.1 | 99.4 | 99.5 | 99.5 | 100.0 |
| STM14_3323 |  | Phosphotransferase system, pseudogene | 300 | TT**T**/TT**G** | 99.7 | 99.7 | 99.7 | 99.8 | 99.7 | 99.7 | 99.4 | 99.7 | 99.7 | 99.7 | 99.7 | 99.7 |
| STM14_3224 |  | Exodeoxyribonuclease VIII | 1534 | **T**AT/**A**AT | 99.7 | 99.7 | 99.7 | 99.7 | 99.4 | 99.7 | 99.7 | 99.6 | 99.6 | 99.7 | 99.7 | 99.5 |
|  |  |  | 1538 | C**T**G/C**A**G | 99.7 | 99.7 | 99.7 | 99.7 | 99.0 | 99.7 | 99.7 | 99.6 | 99.6 | 99.7 | 99.4 | 100.0 |
| STM14_3335 |  | Transposase IS3/IS911 family | 127 | **A**AA/**G**AA | 0 | 0 | 0 | 0 | 0 | 0 | 0 | 0 | 0 | 0 | 0 | 25.0 |
|  |  |  | 135 | CA**G**/CA**A** | 0 | 0 | 0 | 0 | 0 | 0 | 0 | 0 | 0 | 0 | 0 | 25.4 |
|  |  |  | 139 | **C**AC/**T**AC | 0 | 0 | 0 | 0 | 0 | 0 | 0 | 0 | 0 | 0 | 0 | 25.7 |
|  |  |  | 150 | CG**A**/CG**C** | 0 | 0 | 0 | 0 | 0 | 0 | 0 | 0 | 0 | 0 | 0 | 27.3 |
|  |  |  | 153 | AG**C**/AG**T** | 0 | 0 | 0 | 0 | 0 | 0 | 0 | 0 | 0 | 0 | 0 | 27.3 |
|  |  |  | 225 | CG**T**/CG**C** | 0 | 0 | 0 | 0 | 0 | 0 | 0 | 0 | 0 | 0 | 26.3 | 0 |

**Table S3.** Point mutations identified in the genomes of all the *Salmonella* variants and the parental strain sequenced with respect to the genome of the NCBI reference strain ATCC14028s (GenBank CP001363.1).

**Table S3.** (continued)

|  |  |  |  |  | **Mutation Frequency (%)** | | | | | | | | | | | |
| --- | --- | --- | --- | --- | --- | --- | --- | --- | --- | --- | --- | --- | --- | --- | --- | --- |
| **Locus tag** | **Gene** | **Function^a^** | **CDS Position** | **Codon change** | **Parental** | **CI2** | **CI3** | **HT1** | **HT5** | **IC3** | **IC5** | **IC6** | **IC8** | **IC10** | **IT2** | **IT3** |
| STM14_3335 |  | Transposase IS3/IS911 family | 228 | CA**A**/CA**G** | 0 | 0 | 0 | 0 | 0 | 0 | 0 | 0 | 0 | 0 | 28.0 | 27.6 |
|  |  |  | 246 | GA**G**/GA**A** | 0 | 0 | 0 | 0 | 0 | 0 | 0 | 0 | 0 | 0 | 52.1 | 56.2 |
|  |  |  | 249 | GA**A**/GA**G** | 0 | 0 | 0 | 0 | 0 | 0 | 0 | 0 | 0 | 0 | 52.6 | 56.9 |
|  |  |  | 255 | GC**C**/GC**T** | 0 | 0 | 0 | 0 | 0 | 0 | 0 | 0 | 0 | 0 | 54.0 | 58.7 |
|  |  |  | 258 | AT**T**/AT**C** | 0 | 0 | 0 | 0 | 0 | 0 | 0 | 0 | 0 | 0 | 54.5 | 59.6 |
|  |  |  | 264 | CA**G**/CA**A** | 0 | 0 | 0 | 0 | 0 | 0 | 0 | 0 | 0 | 0 | 57.7 | 63.2 |
| STM14_3336 |  | Putative transposase | 15 | TT**C**/TT**T** | 0 | 0 | 0 | 0 | 0 | 0 | 0 | 0 | 0 | 0 | 63.8 | 66.1 |
|  |  |  | 18 | AT**C**/AT**T** | 0 | 0 | 0 | 0 | 0 | 0 | 0 | 0 | 0 | 0 | 63.6 | 66.8 |
|  |  |  | 41 | A**AT**/A**GC** | 0 | 0 | 0 | 0 | 0 | 0 | 0 | 0 | 0 | 0 | 59.3 | 60.8 |
|  |  |  | 51 | GC**C**/GC**A** | 0 | 0 | 0 | 0 | 0 | 0 | 0 | 0 | 0 | 0 | 57.8 | 58.9 |
|  |  |  | 378 | AG**C**/AG**T** | 0 | 0 | 0 | 0 | 0 | 0 | 0 | 0 | 0 | 0 | 0 | 40.8 |
|  |  |  | 387 | AA**T**/AA**C** | 0 | 0 | 0 | 0 | 0 | 0 | 0 | 0 | 0 | 0 | 0 | 38.1 |
|  |  |  | 398 | G**TG**/G**CA** | 0 | 0 | 0 | 0 | 0 | 0 | 0 | 0 | 0 | 0 | 0 | 29.8 |
|  |  |  | 402 | GG**T**/GG**A** | 0 | 0 | 0 | 0 | 0 | 0 | 0 | 0 | 0 | 0 | 0 | 30.4 |
| STM14_4210 | *yrfH* | Heat shock protein Hsp15 | 287 | **A**TG/**-**TG | 0 | 0 | 0 | 0 | 0 | 0 | 0 | 0 | 0 | 0 | 0 | 91.4 |
| STM14_4478 | *rfaJ* | Glycosyl transferase | 742 | AT**A**/AT**-** | 0 | 99.5 | 99.5 | 0 | 0 | 0 | 0 | 0 | 0 | 0 | 0 | 0 |
|  |  |  | 910 | **G**AA/**T**AA | 0 | 0 | 0 | 0 | 99.8 | 0 | 0 | 0 | 0 | 0 | 0 | 0 |
| STM14_4904 | *yneC* | AI-2-degrading protein LsrG | 114 | CG**T**/CG**C** | 99.7 | 99.4 | 99.7 | 99.8 | 99.7 | 99.7 | 99.5 | 99.7 | 99.7 | 99.4 | 99.5 | 100.0 |
| STM14_4922 | *cytR* | Transcriptional regulator, LacI family | 656 | G**A**A/G**T**A | 99.7 | 99.7 | 99.7 | 99.7 | 99.7 | 99.7 | 99.7 | 99.3 | 99.6 | 99.5 | 99.7 | 100.0 |
| STM14_5121 |  | Putative inner membrane protein | 7206 | GT**G**/GT**C** | 99.7 | 99.7 | 99.7 | 99.7 | 99.7 | 99.7 | 99.7 | 99.3 | 99.7 | 99.7 | 99.8 | 100.0 |
|  |  |  | 7243 | **T**TG/**C**TG | 99.7 | 99.8 | 99.5 | 99.8 | 99.7 | 99.7 | 99.5 | 99.3 | 99.7 | 99.8 | 99.8 | 100.0 |
| STM14_5349 | *mgtA* | Magnesium-translocating P-type ATPase | 2234 | C**A**G/C**T**G | 0 | 0 | 0 | 0 | 0 | 99.7 | 0 | 0 | 0 | 0 | 0 | 0 |

^a^, Data extracted from EggNOG. In all cases, P-values were around 0.0.
